# Orthodontic Ceramic Bracket Removal Using Lasers: A Systematic Review

**DOI:** 10.3390/jfb16040123

**Published:** 2025-04-01

**Authors:** Mateusz Michalak, Sylwia Kiryk, Agnieszka Kotela, Kamila Wiśniewska, Jan Kiryk, Jacek Zbigniew Zborowski, Jacek Matys, Maciej Dobrzyński

**Affiliations:** 1 Medical Center of Innovation, Wroclaw Medical University, Krakowska 26, 50-425 Wroclaw, Poland; mateusz.michalak92@gmail.com (M.M.); agnieszka.kotela@student.umw.edu.pl (A.K.); 2 Department of Pediatric Dentistry and Preclinical Dentistry, Wroclaw Medical University, Krakowska 26, 50-425 Wroclaw, Poland; s.roguzinska@gmail.com (S.K.); maciej.dobrzynski@umw.edu.pl (M.D.); 3 Dental Surgery Department, Wroclaw Medical University, Krakowska 26, 50-425 Wroclaw, Poland; jan.kiryk@umw.edu.pl (J.K.); kamila.wisniewska@umw.edu.pl (K.W.); 4 Department of Periodontology, Wroclaw Medical University, Krakowska 26, 50-425 Wroclaw, Poland; jacek.zborowski@umw.edu.pl

**Keywords:** fixed appliance, orthodontic ceramic bracket, removal, laser, teeth

## Abstract

Objective: The aim of this systematic review was to evaluate the effectiveness and safety of various laser systems for debonding ceramic orthodontic brackets compared to conventional mechanical removal methods. The primary outcomes assessed included enamel damage, pulp temperature changes, adhesive remnant index (ARI), and shear bond strength (SBS). Materials and Methods: A systematic search was conducted in November 2024 across the PubMed, Scopus, and Web of Science (WoS) databases following PRISMA guidelines. The initial search yielded 453 records, of which 41 studies met the inclusion criteria for qualitative and quantitative analysis. The risk of bias was assessed using a standardized scoring system, and only studies with accessible full texts were included. Results: The review highlighted significant heterogeneity in laser parameters, measurement protocols, and study methodologies. Among the evaluated lasers, CO_2_ and Er:YAG were the most frequently studied and demonstrated high efficacy in debonding ceramic brackets while maintaining enamel integrity. Sixteen studies assessing SBS reported a reduction from baseline values of 13–23 MPa to clinically acceptable ranges of 7–12 MPa following laser application. ARI was analyzed in 25 studies, with laser-treated groups exhibiting higher scores (2–3), indicating safer debonding with more adhesive remaining on the tooth surface, thereby reducing enamel damage. Pulpal temperature increases were examined in 23 studies, revealing that most laser types, when used within optimal parameters, did not exceed the 5.5 °C threshold considered safe for pulpal health. However, diode and Tm:YAP lasers showed potential risks of overheating in some studies. Conclusions: Laser-assisted debonding of ceramic orthodontic brackets is an effective and safe technique when applied with appropriate laser parameters. CO_2_ and Er:YAG lasers were the most effective in reducing SBS while preserving enamel integrity. However, variations in laser settings, study methodologies, and the predominance of in vitro studies limit the ability to establish standardized clinical guidelines. Further randomized controlled trials (RCTs) are necessary to develop evidence-based protocols for safe and efficient laser-assisted bracket removal in orthodontic practice.

## 1. Introduction

The utilization of ceramic brackets in orthodontic treatment has gained significant popularity due to their superior esthetics and biocompatibility [1]. The bracket system serves as the foundation of fixed orthodontic therapy, with ceramic brackets becoming an increasingly preferred choice among clinicians. Bonding these brackets to teeth necessitates a precise procedure involving composite resin materials, following the conditioning of the enamel surface through acid etching and the application of specific adhesive systems. These brackets are engineered to remain bonded throughout the entire orthodontic treatment, typically lasting two to three years. However, the debonding of these brackets presents a critical clinical challenge. Ceramic brackets exhibit higher bond strength compared to metal brackets and are additionally more brittle and susceptible to fracture, thereby increasing the risk of enamel damage during their removal [2,3].

Over the years, several methods have been developed for the debonding of ceramic brackets, including mechanical debonding (using specialized pliers), electrothermal debonding, chemical debonding, and ultrasonic debonding [2,4,5,6]. Currently, mechanical debonding is the most commonly employed technique, wherein brackets are typically removed using specialized pliers [4]. Despite its widespread use, this conventional approach raises several clinical concerns related to both efficiency and safety. Studies have reported varying degrees of enamel damage during the mechanical debonding process [2,7]. A significant challenge in debonding is managing adhesive residues that remain on the tooth surface after bracket removal. The mechanical removal of these residues often necessitates the use of rotating instruments, which can compromise the integrity of the enamel structure [7,8,9]. Additionally, patients may experience pain and discomfort when ceramic brackets are removed using mechanical methods [10]. Ultrasonic debonding presents an alternative approach, as it reduces the adhesive bond strength of the brackets, thereby enhancing the safety of the procedure. However, this method generally requires a longer procedure time, which may be inconvenient for patients [4,5]. Chemical debonding has also been explored; however, it does not significantly reduce shear bond strength (SBS) [3]. Electrothermal debonding has been investigated as another alternative, demonstrating safety by significantly lowering SBS levels without causing damage to the enamel or pulp [6].

**Figure 1:**
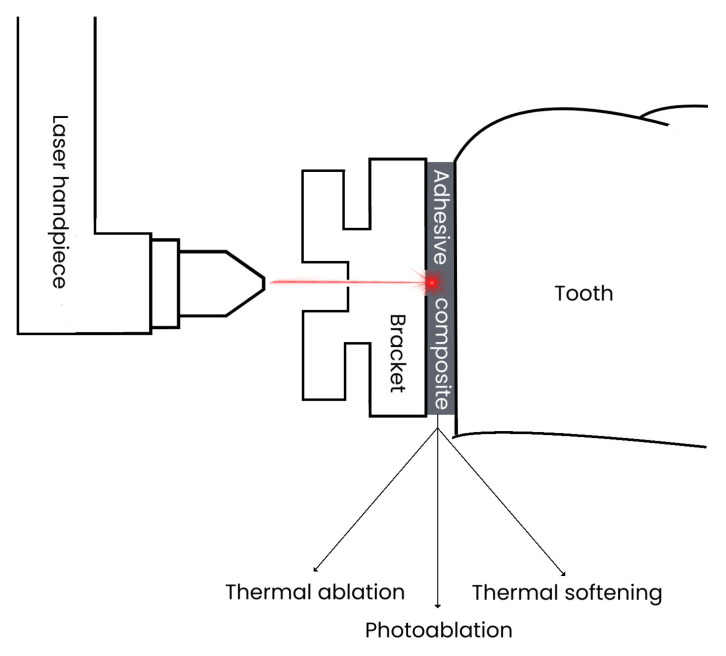
Concept describing the mechanism of debonding using lasers.

In response to these challenges, researchers are investigating novel debonding methods, with laser technology emerging as a promising alternative for bracket removal. Current studies focus on identifying optimal laser parameters that can effectively debond brackets while minimizing potential damage to the enamel surface. Various laser systems and settings are being evaluated to establish the most efficient and safe protocols for ceramic bracket removal [11]. Laser energy facilitates the removal of adhesive resin from the tooth surface through three primary mechanisms: thermal softening, thermal ablation, and photoablation [2]. Specifically, laser technology provides an effective method for orthodontic bracket removal through its thermal softening mechanism [12]. The laser energy is converted into heat, causing the adhesive material to soften and weaken, thereby allowing the bracket to be naturally released from the tooth surface [2,13,14,15,16] (see Figure 1).

The effective removal of orthodontic brackets using lasers necessitates the maintenance of several critical factors, including the preservation of enamel integrity, thermal regulation within physiological limits, and the minimization of residual adhesive material, as assessed by the adhesive remnant index (ARI) [2,17,18,19,20]. Despite its effectiveness, laser debonding presents limitations related to heat generation within the tooth structure. An increase in temperature of 5.5 °C can cause irreversible damage to the pulp tissue [2,21,22,23]. Furthermore, a temperature rise of 6 °C may damage the periodontal ligament [24], and an increase of up to 10 °C can result in bone damage [25,26]. Therefore, precise calibration of laser parameters—such as wavelength, power output, and exposure duration—is essential for achieving optimal bracket removal while safeguarding both the enamel surface and the deeper dental tissues from potential thermal or structural damage [2,27].

The aim of this systematic review is to evaluate the effectiveness of different laser wavelengths in debonding orthodontic ceramic brackets compared to traditional methods, with particular emphasis on identifying parameters that minimize enamel damage while maintaining clinical efficiency. Upon analyzing relevant studies on the use of lasers for ceramic bracket debonding, it became evident that the absence of a comprehensive systematic review in this area represents a significant opportunity to consolidate existing evidence. This review seeks to provide clinicians with evidence-based recommendations for the implementation of laser-assisted debonding techniques.

## 2. Materials and Methods

### 2.1. Focused Question

The systematic review followed the PICO framework [28] as follows: In the case of orthodontic ceramic bracket debonding (population), will the use of lasers (investigated condition) be more effective (outcome) in comparison to conventional methods (comparison condition)?

### 2.2. Protocol

**Figure 2:**
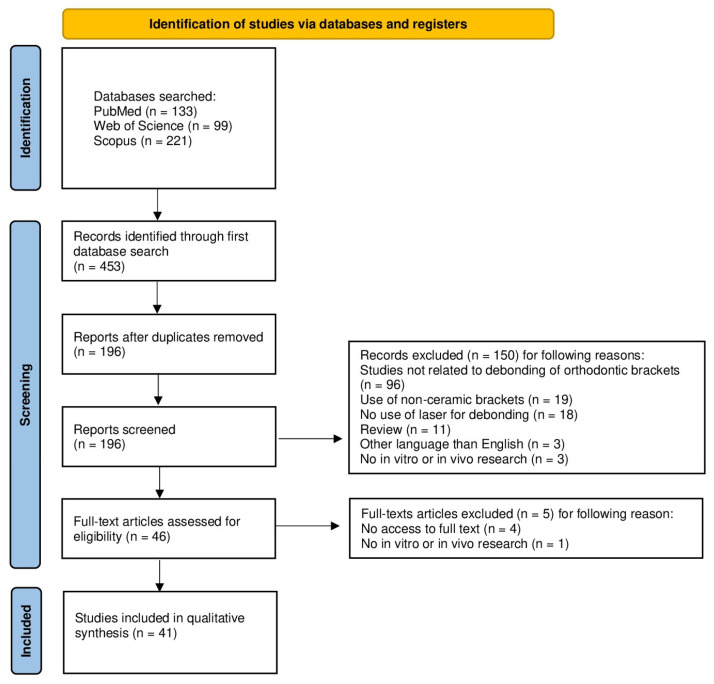
The PRISMA 2020 flow diagram.

The selection process for the articles included in the systematic review was carefully outlined following the PRISMA flow diagram [29] (see Figure 2). The systematic review was registered on the Open Science Framework under the following link: https://osf.io/sh2xw/ (accessed on 18 February 2025).

### 2.3. Eligibility Criteria

The researchers agreed to include only the articles that met the following criteria [30,31,32,33,34,35,36,37]:

• Laser debonding;
• Use of ceramic brackets;
• Use of all types of lasers;
• In vitro and in vivo studies;
• Studies published in English;
• Full-text availability.

The exclusion criteria the reviewers agreed upon were as follows [30,31,32,33,34,35,36,37]:

• Use of metal brackets;
• Use of forceps or ultrasound to debond brackets;
• Review articles;
• Duplicated publications.

No restrictions were applied with regard to the year of publication.

### 2.4. Information Sources, Search Strategy, and Study Selection

In November 2024, the PubMed, Scopus, and Web of Science (WoS) databases were searched for articles that met the specified inclusion criteria. To identify studies on laser debonding of ceramic orthodontic brackets, the search was refined using specific keywords. For PubMed, we used (orthodontic [Title/Abstract]) AND (brackets [Title/Abstract]) AND (laser [Title/Abstract]) AND (removal [Title/Abstract]); (orthodontic [Title/Abstract]) AND (brackets [Title/Abstract]) AND (laser [Title/Abstract]) AND (debonding [Title/Abstract]). For WoS, we used AB = (orthodontic AND brackets AND laser AND removal); AB = (orthodontic AND brackets AND laser AND debonding). For Scopus, we used TITLE-ABS-KEY (orthodontic) AND TITLE-ABS-KEY (brackets) AND TITLE-ABS-KEY (laser) AND TITLE-ABS-KEY (removal); TITLE-ABS-KEY (orthodontic) AND TITLE-ABS-KEY (brackets) AND TITLE-ABS-KEY (laser) AND TITLE-ABS-KEY (debonding). All searches adhered to the predefined eligibility criteria and only articles with accessible full-text versions were included.

### 2.5. Data Collection and Data Items

Five reviewers (J.K., A.K., K.W., M.M. and S.K.) meticulously selected the articles that met the inclusion criteria. The extracted data were then entered into a standardized Excel file.

### 2.6. Assessing Risk of Bias in Individual Studies

In the preliminary phase of study selection, the authors independently reviewed the titles and abstracts of each study to minimize the risk of reviewer bias. They assessed the level of agreement among reviewers using Cohen’s κ test [38]. Any disagreements regarding the inclusion or exclusion of studies were resolved through discussion.

### 2.7. Quality Assessment

Two independent evaluators (J.M. and M.D.) assessed the procedural quality of each study included in the analysis. The evaluation criteria encompassed randomization, a minimum group size of 10 samples, the presence of a control group, sample size calculation, and a detailed description of laser parameters and the debonding protocol. Studies were scored on a scale of 0 to 6 points, with higher scores indicating better study quality. The risk of bias was classified as follows: 0–2 points indicated a high risk, 3–4 points a moderate risk, and 5–6 points a low risk. Any discrepancies in scoring were resolved through discussion until a consensus was reached [30,31,32,33,34,35,36,37].

## 3. Results

### 3.1. Study Selection

The initial search of the electronic databases yielded 453 records. After removing 257 duplicates, 196 unique records remained for abstract screening. During this phase, 150 articles were excluded for the following reasons: 96 studies were unrelated to the debonding of orthodontic brackets, 19 used brackets made of materials other than ceramic, 18 did not utilize lasers for debonding, 11 were review articles, 3 were published in languages other than English, and 3 did not involve in vitro or in vivo research. This left 46 articles for full-text review. Of these, five were excluded: one was a theoretical study without in vitro or in vivo research, and access to four publications was unavailable. Consequently, 41 articles were selected for both qualitative and quantitative analyses (see Figure 2).

### 3.2. General Characteristics of the Included Studies

The studies included in this systematic review exhibited heterogeneity in the types of lasers used for debonding ceramic orthodontic brackets, the parameters assessed, and the outcomes related to enamel and pulp. The primary objective of most studies was to evaluate the efficacy of various lasers, including CO_2_ [39,40,41,42,43,44,45,46,47,48,49], Tm:YAP [50,51,52,53], Nd:YAG [12,44,54,55], Er:YAG [3,13,56,57,58,59,60,61,62,63,64,65,66,67,68], Er,Cr:YSGG [56,60,63,69,70], and diode lasers [3,53,71,72,73,74,75] with different wavelengths. The studies primarily focused on changes in pulp temperature [13,39,40,41,43,45,46,47,50,51,52,53,55,56,57,59,65,66,70,71,74,75,76], shear bond strength [3,39,40,42,43,47,48,49,50,54,55,60,62,63,66,67,68,73,75], and adhesive residues remaining after the procedure [3,13,39,40,41,42,43,44,47,48,49,54,60,61,62,64,65,66,67,68,69,70,72,73,74,75] (see Supplementary Table S1).

#### 3.2.1. CO_2_ Laser Debonding

Among the authors who utilized the CO_2_ laser, all unanimously agreed that it is an effective and safe method for debonding ceramic brackets [39,40,41,42,43,44,45,46,47,48,49]. Additionally, three studies concluded that its use significantly reduces the risk of enamel surface damage [44,47,49]. Several researchers highlighted the importance of the adhesive material used [40,43,48]. Arima et al. [40] and Saito et al. [43] incorporated thermally expanded microcapsules into a conventional composite, and both concluded that this modification yields the best results for debonding with a CO_2_ laser. However, while Saito et al. [43] found no difference in the adhesive remnant index (ARI) value, Arima et al. [39], who tested different proportions, reported improved ARI results with a 25% microcapsule content. Mimura et al. [48] compared a material containing BIS-GMA resin to MMA resin without filler, finding significantly better results with the MMA resin. A decrease in shear bond strength (SBS) was observed only by Macri et al. [40].

#### 3.2.2. Er:YAG (Erbium–Yttrium, Aluminum, Garnet) Laser Debonding

The use of the Er:YAG laser has also been shown to be effective, with authors consistently agreeing on its efficacy and safety for debonding ceramic brackets [3,13,56,57,58,59,60,61,62,63,64,65,66,67,68]. However, several researchers emphasize the importance of laser parameters, demonstrating consistent findings [57,64,65,67]. Hamadah et al. [64] identified a pulse duration of 100 to 300 milliseconds as optimal. Nalbantgil et al. [65], utilizing a pulse duration of 300 milliseconds, further recommended a power setting of 4 W, an exposure time of 6 s, and the use of a scanning mode for optimal results. Yilanci et al. [57], applying exposure times of 4–6 s, observed that while longer exposure increases temperature, it remains safe within this duration. Oztoprak et al. [67] confirmed that employing a scanning mode significantly reduces shear bond strength (SBS), facilitating safer debonding.

#### 3.2.3. Diode Laser Debonding

The use of diode lasers for debonding has not received as much enthusiasm from researchers as CO_2_ or Er:YAG lasers. Four publications report positive outcomes, stating that diode lasers shorten working time and reduce the risk of enamel damage [71,72,73,74]. Notably, three of these studies used a laser with a 445 nm wavelength, with Steffen Stein as the lead author in each case [71,72,73]. Other studies, however, present varying results. Feldon et al. [75] observed a significant decrease in shear bond strength (SBS) only when using monocrystalline brackets. Nalbantgil et al. [65], who also studied monocrystalline brackets and compared different lasers, concluded that the Er:YAG laser is more effective and safer than the 980 nm diode laser. Dostalová et al. [53] found that the 808 nm diode laser could heat the tooth surface to as high as 114 °C without causing bracket detachment, raising concerns about potential thermal effects on dental tissues.

#### 3.2.4. Er,Cr:YSGG (Erbium, Chromium–Yttrium, Scandium, Gallium, Garnet) Laser Debonding

Authors comparing different laser types concluded that the Er,Cr:YSGG laser is equally as effective and safe as the Er:YAG laser [56,60,63]. However, Hoteit et al. [63] cautioned that improper parameter settings for both lasers could lead to enamel damage, though they did not specify the power and exposure time used in their studies. Rao et al. [69] determined that power settings between 4.5 and 6 W are completely safe for debonding. Abdulaziz et al. [70], using a power setting of 4 W, observed that operating in scanning mode resulted in a smaller temperature increase, further enhancing the safety of the procedure.

#### 3.2.5. Nd:YAG (Neodymium–Yttrium, Aluminum, Garnet) Laser Debonding

The Nd:YAG laser was used by only four researchers, yet all reached the same conclusion: it is a fast and painless method for the patient. Its application reduces both shear bond strength (SBS) and the adhesive remnant index (ARI) while minimizing the risk of enamel damage. Hayakawa et al. [55] investigated the debonding process using various types of adhesives and brackets. Their findings indicate that the Nd:YAG laser is effective regardless of the adhesive used, although monocrystalline brackets are more easily debonded.

#### 3.2.6. Tm:YAP (Thulium–Yttrium, Aluminum, Perovskite) Laser Debonding

The use of the Tm:YAP laser warrants special attention due to the significant variation in its effects depending on the applied parameters. Dostalová et al. [52] studied its performance in 2011 at a power range of 1–2 W and found that irradiated brackets were removed with most of the adhesive, making it a useful tool for debonding. However, in a follow-up study in 2012, they observed that while shear bond strength (SBS) decreases at 1 W, using 4 W power leads to a significant increase in SBS [51]. Demirkan et al. [50] provided a detailed analysis of temperature changes associated with Tm:YAP laser use. They found that a safe temperature increase was achieved with 2.5–3 W power under the following conditions: 7 s at 3 W in scanning mode, 7 s at 2.5 W, and 10 s at 3 W in non-scanning mode. Dostalová et al. [53] further investigated the laser’s thermal effects and concluded that irradiation at 1–2 W for more than 60 s or without proper cooling can cause irreversible changes in the dental pulp.

### 3.3. Main Study Outcomes

The main outcomes evaluated in the studies included shear bond strength (SBS), assessed by 16 research groups [3,39,40,42,43,47,48,49,60,62,63,66,67,68,73,75]. Twenty-five studies assessed ARI, confirming that laser-assisted debonding shifts adhesive failure from the enamel–adhesive interface toward the bracket–adhesive interface, reducing enamel damage risk [3,13,39,40,41,42,43,44,48,49,55,60,61,62,64,65,66,67,68,69,70,72,73,74,75]. Temperature increase was evaluated in twenty-one studies, with all findings confirming that laser use remains within the 5.5 °C safety threshold, ensuring pulp vitality [13,39,40,41,43,45,46,47,48,50,51,52,56,57,59,65,66,70,71,74,76]. CO_2_ and Nd:YAG lasers exhibited the highest temperature increases, while Er:YAG and Er,Cr:YSGG lasers showed the lowest thermal effects, making them preferable for temperature-sensitive applications.

Various laser types were used, including CO_2_ [39,40,41,42,43,44,45,46,47,48,49], five researchers used an Er,Cr:YSGG laser [56,60,63,69,70], twelve an Er:YAG laser [13,57,58,59,60,61,62,64,65,66,67,68], three a Nd:YAG laser [12,54,55], two a fiber laser [50,76], seven a diode laser [3,53,71,72,73,74,75], and three researchers used a YAP laser [51,52,53], with Er:YAG and Er,Cr:YSGG lasers emerging as the most effective options.

The studies included different bracket materials, ceramic [13,43,46,51,52,53,56,59,63,64,75], monocrystalline [3,12,41,47,55,57,58,70,75], polycrystalline [12,40,41,42,44,45,48,49,50,54,55,61,62,65,66,67,68,71,72,73,74,76], and zirconia brackets [39], and demonstrated that monocrystalline and zirconia brackets require higher debonding forces (see Table 1).

### 3.4. Quality Assessment

Among the articles included in the review, twelve studies [3,39,41,47,55,56,62,66,67,70,72,73] were rated as high quality, achieving scores of between 5 and 6 out of 6. Twenty-four studies [12,13,42,43,45,48,49,50,51,52,53,56,58,59,60,63,64,65,68,69,71,74,75,76] were identified as having a moderate risk of bias with scores ranging from 3 to 4. Five of the studies [44,46,54,57,61] included in this review were classified as low quality (see Table 2).

## 4. Discussion

The objective of this systematic review was to evaluate the effectiveness and safety of laser-assisted debonding of ceramic orthodontic brackets. The lasers investigated in the included studies were the CO_2_ laser [39,40,41,42,43,44,45,46,47,48,49], Er,Cr:YSGG laser [56,60,63,69,70], Er:YAG laser [3,13,56,57,58,59,60,61,62,63,64,65,66,67,68], Nd:YAG laser [12,44,54,55], fiber laser [50,76], diode laser [3,53,71,72,73,74,75], and Tm:YAP laser [50,51,52,53]. The findings suggest that laser-assisted debonding generally leaves more adhesive residue on the tooth surface compared to conventional debonding methods [3,41,48,49,67,68,72,73]. However, laser irradiation effectively and safely reduces shear bond strength (SBS), provided that appropriate parameter settings—such as laser power, exposure time, and irradiation technique—are utilized [3,39,40,42,43,47,48,49,60,62,63,66,67,68,72,75]. The increase in pulp temperature during laser irradiation varies based on the type of laser, power output, exposure duration, and irradiation method employed [13,39,40,41,43,45,46,47,50,51,52,53,55,56,57,59,65,66,70,71,74,75,76]. These findings emphasize the importance of optimizing laser parameters to achieve efficient debonding while minimizing the risk of thermal damage to dental tissues.

Laser debonding of orthodontic brackets poses a potential risk of thermal damage to the dental pulp [2]. A temperature increase of no more than 5.5 °C is generally considered safe and unlikely to cause irreversible pulp damage [66]. Therefore, selecting an appropriate laser type and optimizing its operational parameters are crucial for ensuring a safe debonding procedure. Of the 41 studies analyzed, 23 specifically evaluated the temperature increase during laser-assisted debonding of ceramic brackets [13,39,40,41,43,45,46,47,50,51,52,53,55,56,57,59,65,66,70,71,74,75,76], while 18 studies did not include temperature measurements [3,12,42,44,48,49,54,58,60,61,62,63,64,67,68,69,72,73]. The results indicate that, in most cases, the use of CO_2_, Er:YAG, diode, Er,Cr:YSGG, Nd:YAG, Tm:YAP, Tm:fiber, and ytterbium fiber lasers did not cause a pulp temperature increase exceeding the 5.5 °C threshold, suggesting their relative safety for debonding procedures [13,39,40,41,43,45,46,50,51,52,55,57,59,65,66,70,71,74,75,76]. However, temperature elevation was directly correlated with increased laser power. While most tested lasers remained within the safe range, one study reported pulp-damaging temperatures when using a diode laser and a Tm:YAP laser [53]. For CO_2_ lasers, the mode of irradiation significantly influenced temperature rise. Irradiation in the pulse mode at 5–10 W for 3–5 s [40] and at 188 W for 5 s [41] resulted in a lower temperature increase than in the continuous wave mode at 7 W for 6 s [39]. Additionally, in the same study, when a CO_2_ laser was set to 3 W, the temperature increase was lower in the super pulse mode (+2.1 °C) than in the normal pulse continuous wave mode (+2.7 °C) [46]. Abdulaziz et al. [70] reported that using a CO_2_ laser at 4 W in the scanning mode resulted in a lower temperature increase. Similarly, Demirkan et al. [50] found that temperature elevation was reduced in the scanning mode, but only under specific conditions: 2.5 W with a 10 s exposure and 3 W with a 7 s exposure. These findings highlight the importance of optimizing laser parameters, particularly power settings, exposure duration, and irradiation mode, to mitigate thermal risks while ensuring effective bracket debonding.

Reducing shear bond strength (SBS) between the tooth and the ceramic orthodontic bracket is a critical factor in achieving safe and efficient debonding while minimizing the risk of enamel damage. An analysis of 41 published studies revealed that 16 studies specifically evaluated SBS during laser-assisted debonding of ceramic brackets [3,39,40,42,43,47,48,49,60,62,63,66,67,68,73,75], whereas 25 studies did not include SBS measurements [12,13,41,44,45,46,50,51,52,53,54,55,56,57,58,59,61,64,65,69,70,71,72,74,76]. The findings demonstrated that laser exposure effectively reduces SBS compared to conventional debonding methods. For CO_2_ lasers, optimal parameters included a power setting of 5–10 W with an exposure duration of 3–6 s in a continuous wave mode [39,40,42,43,47,48,49]. In contrast, the Er:YAG laser yielded the best results at a power of 3–4 W with an exposure duration of 6–9 s, particularly when applied using a scanning motion [62,66,67,68]. Studies reported that laser application reduced SBS from baseline values of 13–23 MPa to 7–12 MPa, with higher power settings and longer exposure durations (within safe limits) leading to greater SBS reductions [40,49,62,66,67,68]. Most studies concluded that the reduced SBS values remained within clinically acceptable ranges (5–10 MPa) for safe bracket removal while mitigating the risk of enamel damage [40,42,62,66,67,68]. Furthermore, the application of a scanning motion proved to be more effective than static irradiation across different laser types, ensuring a more uniform energy distribution and reducing the risk of localized overheating [62,66,67]. These findings emphasize the importance of optimizing laser parameters to achieve efficient debonding while preserving enamel integrity.

The adhesive remnant index (ARI) is a crucial parameter that quantifies the amount of adhesive residue remaining on the tooth surface after bracket debonding, playing a key role in assessing the effectiveness and safety of different debonding techniques [2]. Among the 41 analyzed studies, 25 specifically evaluated ARI [3,13,39,40,41,42,43,44,47,48,49,60,61,62,64,65,66,67,68,69,70,72,73,74,75], while 16 did not include ARI measurements [12,45,46,50,51,52,53,54,55,56,57,58,59,63,71,76]. Most studies employed a standardized 4-point ARI scale, where Score 0 indicated no adhesive remaining on the tooth surface and Score 3 represented complete adhesive retention. A consistent trend emerged across laser types, with laser-treated groups exhibiting a higher frequency of Scores 2 and 3 [41,48,67,68,72,73], suggesting more adhesive remained on the tooth surface compared to control groups, which showed a higher prevalence of Scores 0 and 1 [3,41,49]. Studies on CO_2_ [41,48,49], Er:YAG [61,62,64,66,67,68], diode [3], and Nd:YAG [54] lasers confirmed that bond failure predominantly occurred at the bracket–adhesive interface rather than the enamel–adhesive interface, reducing the risk of enamel damage. In contrast, conventional debonding methods showed a higher occurrence of Scores 0 and 1 [41,49,66,67,68,69,70], indicating bond failure often occurred at the enamel–adhesive interface, increasing the potential for enamel microfractures. Several studies [3,13,41,42,49,67,68] concluded that the higher ARI scores associated with laser debonding offer clinical benefits, as they reduce enamel damage risk, improve debonding safety by shifting failure to the bracket–adhesive interface, and enhance tooth surface preservation by minimizing the need for aggressive adhesive removal. The consistency of these findings suggests that laser-assisted debonding provides a safer and more protective alternative to conventional mechanical methods, making it a valuable tool in contemporary orthodontic practice.

The reviewed research on laser-assisted debonding of ceramic brackets demonstrates significant heterogeneity in methodology and measurement protocols across studies, posing challenges in drawing definitive clinical conclusions. One of the primary limitations is the predominance of in vitro studies over clinical trials, with only a single in vivo investigation available [58]. Additionally, variability in the type of teeth used for experimentation introduces further inconsistency, as the majority of studies (28) utilized human teeth, whereas 7 studies used bovine teeth, and 2 studies incorporated both types. The lack of uniformity in laser settings, application techniques, and measurement protocols for shear bond strength (SBS), adhesive remnant index (ARI), and temperature assessment further complicates direct comparisons across studies. The diversity in research approaches and the absence of standardized protocols significantly limit the ability to formulate universal clinical recommendations. These limitations underscore the urgent need for more extensive, well-designed randomized clinical trials (RCTs) with larger sample sizes and standardized methodologies to ensure reproducibility and clinical applicability. Future studies should focus on establishing evidence-based guidelines for laser parameters, application techniques, and safety thresholds to optimize debonding efficiency while minimizing risks. To facilitate the integration of laser-assisted debonding into routine orthodontic practice, further investigations should aim to develop a standardized protocol that ensures both effectiveness and patient safety.

## 5. Conclusions

The systematic review of 41 studies on laser-assisted ceramic bracket removal has demonstrated promising effectiveness across multiple laser types, particularly CO_2_ and Er:YAG lasers, which were the most frequently studied and reported as safe and efficient for debonding. The majority of studies confirmed that laser-assisted techniques provide a reliable alternative to conventional mechanical methods while minimizing the risk of enamel damage. Shear bond strength (SBS) was evaluated in 16 studies, revealing a significant reduction from baseline values of 13–23 MPa to clinically acceptable ranges of 7–12 MPa post-laser application, facilitating safer bracket removal. Additionally, 25 studies assessed the adhesive remnant index (ARI), with laser-treated groups consistently exhibiting higher ARI scores (2–3), indicating that bond failure primarily occurred at the bracket–adhesive interface rather than the enamel–adhesive interface, thereby reducing the risk of enamel microfractures compared to conventional methods. Temperature monitoring, conducted in 23 studies, confirmed that carefully optimized laser parameters can maintain pulpal temperature increases within safe limits, preventing irreversible thermal damage. However, despite these positive findings, precise laser parameter settings remain critical for ensuring both efficiency and safety, particularly in preventing excessive temperature elevation that could compromise pulpal health. Given the heterogeneity in methodologies and the predominance of in vitro studies, further randomized clinical trials (RCTs) with standardized protocols are essential to establish evidence-based guidelines for optimal laser parameters and application techniques. This will facilitate the safe and effective integration of laser-assisted debonding into routine orthodontic practice while maximizing patient safety and treatment outcomes.

## Figures and Tables

**Table 1:** Detailed characteristics of studies.

Authors	Laser Parameters	Protocol of Debonding	Brackets	SBS	Results ARI	Temperature Increase
Khalil [3]	Diode laser (Simpler, Doctor Smile, Italy) with continuous mode Power: 4 W Wavelength: 980 nm Tip diameter: 300 μm Time: 12 s Er:YAG laser (Pluser, Doctor Smile, Brendola, Italy) Power: 4 W Wavelength: 2940 nm Tip diameter: 1 mm	Shear testing of the brackets was performed with a universal testing machine, and then ARI assessment and scanning electron microscopy were used to evaluate the enamel microstructure.	Brackets: monocrystalline ceramic brackets (Perfect Clear, Hubit, Uiwang-si, Republic of Korea) Adhesive: GC Ortho Connect adhesive (GC Ortho Connect, GC Orthodontics, Breckerfeld, Germany).	Mean shear bond strength Group I (control): 14.99 MPa Group II (chemical-aided debonding): 14.25 MPa Group III (ultrasonic-aided debonding): 11.17 MPa Group IV (diode laser-aided debonding): 11.13 MPa Group V (Er:YAG laser-aided debonding): 9.39 MPa	Statistically significant higher ARI was found solely in group V when compared to group I, group II, group III, and group IV. No other significant differences were found between the groups with regard to ARI.	No data
Tocchio [12]	Nd:YAG laser XeCI excimer (Hypcrex- 400, Lumonics, Kanata, ON, Canada) Wavelengths: 248 nm, 308 nm, 1060 nm Power: 8 W Pulse energy: 21 mJ	The brackets were debonded by exposing their labial surfaces to XeCI excimer laser light with wavelengths of 248 nm, 308 nm, and 1060 nm, with light power densities ranging from around 3 to 33 W/cm 2 under an externally induced stress of either 0.8 MPa or 0 MPa.	Brackets: single crystal alumina (sapphire) brackets (Starfire, “A” Company, San Diego, CA, USA) and polycrystalline alumina brackets (Transcend, Unitek, Monrovia, CA, USA) Adhesive: no data	No data	No data	No data
Grzech-Leśniak [13]	Er:YAG laser (Morita, Irvine, CA, USA) Time: 6 s Distance: 1 mm (groups 1, 2), 2 mm (group 3) Wavelength: 2940 nm Power: 3.4 W Energy: 170 mJ Frequency: 20 Hz Pulse duration: 300 ls Tip diameter: 0.8 mm Level of air/fluid: 3 mL/s	Three different laser application methods for bracket debonding were used.	Brackets: metal (Victory Series; 3M Unitek, Monrovia, CA, USA) and ceramic brackets (Inspire-ICE; Ormco, Orange, CA, USA) Adhesive: Transbond XT (3M Unitek, Monrovia, CA, USA). The Er:YAG laser was used to irradiate the brackets.	No data	Mean ranks: Group 1: 25.80 Group 2: 22.03 Group 3: 21.20	The mean temperature gradient Group 1: 1.29 ± 0.42 °C Group 2: 1.78 ± 0.60 °C Group 3: 0.83 ± 0.43 °C All groups: 1.30 ± 0.62 °C
Arima [39]	CO_2_ laser 10,600 nm Time: 3–6 s Distance: in contact with labial surface of the bracket Power: 5 or 7 W Beam diameter: 0.15 mm Continuous wave (CW)	Irradiation for 3, 4, 5, and 6 sec at an intensity of 5 W or 7 W with (C) and without (NC) air cooling. SBS measured 10 min after irradiation.	Zirconium brackets (COBY, Biodent, Tokyo, Japan) with base area 12.4 mm^2^ Adhesive: Transbond XT (3M Unitek, Monrovia, CA, USA) + bonding agent containing microcapsules (0–30 wt%)	Change compared to the control group: 30% of agent containing microcapsules = −6.8 MPa 25% = −0.17-fold 20% = −0.39-fold 10% = −0.89-fold 0% = −0.81-fold	0% of microcapsule content: Score 0 × 4 Score 1 × 2 10% Score 0 × 1 Score 1 × 5 20% Score 0 × 3 Score 1 × 3 25% Score 0 × 5 Score 1 × 2 30% Score 0 × 4 Score 1 × 2	Maximum temp. increase: 5 s, 7 W, C = 5.3 °C 6 s, 7 W, C = 5.9 °C 5 s, 7 W, NC = 6.6 °C 6 s, 7 W, NC = 7.4 °C
Macri [40]	CO_2_ laser 10,600 nm Time: 3 or 5 s Pulse duration: 0.001 or 0.003 s Distance: 4 mm Power: 5, 8, or 10 W	SBS was measured immediately after laser irradiation.	Polycrystalline bracket (Fascination, Dentaurum, Ispringen, Germany) Adhesive: Transbond XT (3M/Unitek, Monrovia, CA, USA)	10 W, of 0.01 s pulse = 7.33 (1.89) MPa 8 W, of 0.01 s pulse = 9.04 (3.26) MPa 5 W, of 0.01 s pulse = 10.56 (3.47) MPa 5 W, of 0.03 s pulse = 11.72 (5.42) MPa	Mean: 10 W, of 0.01 s pulse = 2.53 8 W, of 0.01 s pulse = 1.66 5 W, of 0.01 s pulse = 2.26 5 W, of 0.03 s pulse = 2.46	Mean increase: 10 W, of 0.01 s pulse; 8 W, of 0.01 s pulse; 5 W, of 0.01 s pulse; 5 W, of 0.03 s pulse; Less than 5.5 °C Irradiation with other combinations of parameters above 5.5 °C
Ahrari [41]	CO_2_ laser 10,600 nm Time: 5 s Distance: 5 mm Power: 188 W Pulse duration: 500 μs Beam diameter: 1 mm Surface area cm^2^ Frequency: 400 Hz	Brackets were debonded with pliers 3 s after irradiation.	Polycrystalline brackets Fascination (Dentaurum, Ispringen, Germany) or monocrystalline brackets Inspire Ice (Ormco, Orange, CA, USA) Adhesive: Transbond XT (3M Unitek, Monrovia, CA, USA)	No data	Polycrystalline brackets Score 0 × 1 Score 1 × 5 Score 2 × 9 Score 3 × 5 Monocrystalline brackets Score 0 × 2 Score 1 × 1 Score 2 × 3 Score 3 × 14	Mean increase Polycrystalline brackets 3.9 ± 0.32 °C Monocrystalline brackets 4.4 ± 0.5 °C
Matos [42]	CO_2_ laser 10,600 nm Time: 3 s Distance: 4 mm Power: 10 W Pulse duration: 0.01 s	SBS testing was performed right after laser irradiation	Polycrystalline brackets Fascination (Dentaurum, Ispringen, Germany) or monocrystalline brackets Inspire Ice (Ormco, Orange, CA, USA) Adhesive: Transbond XT (3M Unitek, Monrovia, CA, USA) or Z250 (3M ESPE, Dental Products Division, St. Paul, MN, USA)	Polycrystalline brackets + Transbond XT = 0.92 (0.18) MPa Polycrystalline brackets + Z250 = 0.28 (0.7) MPa Monocrystalline brackets + Transbond XT = 3.45 (0.68) MPa Monocrystalline brackets + Z250 = 3.52 (1.04) MPa	Mean Polycrystalline brackets + Transbond XT = 1.6 (1.3) Polycrystalline brackets + Z250 = 2.66 (0.48) Monocrystalline brackets + Transbond XT = 1.2 (1.08) Monocrystalline brackets + Z250 = 1.86 (0.99)	No data
Saito [43]	CO_2_ laser (Opelaser Pro, Yoshida, Tokyo, Japan) Distance: in contact with the labial face of a bracket head Beam diameter: 0.5 mm Power: 3 W Time: few seconds	Bonding materials with different microcapsule contents (0, 30, and 40 weight percent) were used to bond ceramic brackets to bovine permanent mandibular incisors. The bond strengths were assessed following laser irradiation for 4, 5, and 6 s and compared to groups that were not laser-treated. A measurement of the pulp chamber’s temperature during laser irradiation was then performed.	Brackets: ceramic brackets Adhesive: experimentally produced 4-META/MMA-TBB resin orthodontic adhesives (Orthomite SuperBond, Sun Medical, Moriyama, Japan) containing 30 and 40 wt% thermal expansion microcapsules, which expanded 70-fold upon heating to 80 °C (Matsumoto Microsphere F-36D, Matsumoto Yushi- Seiyaku, Osaka, Japan) in the polymer powder.	Shear bond strengths around 18 MPa without laser irradiation did not change with laser irradiation for 4–6 s when the adhesive did not contain microcapsules.	No significant difference in ARI score after debonding was detected between with and without CO_2_ laser irradiation, but there were many scores of 0 and 1.	Temperature increases in the pulp chamber for each irradiation were less than 4.3 °C.
Strobl [44]	CO_2_ laser 10,600 nm Time: 2 s Power: 7–14 W Nd:YAG laser 1060 nm Time: 5 s	Brackets were debonded with bracket removal fork after irradiation.	Polycrystalline alumina (A12OD (Transcend, Unitek/3M, Monrovia, CA, USA)) and 30 monocrystalline (sapphire) alumina brackets (Starfire, A Company/Johnson & Johnson, San Diego, CA, USA). Adhesive: Concise (3M, Minneapolis, MN, USA).	No data	ARI for brackets debonded without laser Polycrystalline: Score 1 × 24 Score 2 × 3 Score 3 × 0 Score 4 × 2 Score 5 × 1 Monocrystalline: Score 1 × 16 Score 2 × 3 Score 3 × 1 Score 4 × 0 Score 5 × 0	No data
Tsun Ma [45]	CO_2_ laser 10,600 nm Time: 1, 2, 3 s Distance: as close as possible to the labial surface of the ceramic bracket Power: 18 W Beam diameter: 1 mm Waveguide length: 1 m	Debonding force was applied during the laser irradiation (1, 2, 3 s) using modified pliers with laser waveguide. The ceramic bracket was removed from the tooth as soon as the adhesive softening temperature was reached.	Polycrystalline alumina orthodontic brackets (Transcend 6000, Unitek/3M), Adhesive: Transbond, Unitek/3M)	No data	No data	Human teeth: 1 s, +0.91 (°C) 2 s, +1.74 (°C) 3 s, +2.67 (°C) Bovine teeth: 1 s, +1.65 (°C) 2 s, +3.31 (°C) 3 s, +5.15 (°C)
Akihito Obata [46]	2 super pulse and 1 continuous wave normal pulse CO_2_ laser 10,600 nm Power: 2, 3, 4 W Super pulse: Pulse width: 1–500 milliseconds Pulse width: 200–800 microseconds Normal pulse: Pulse width: 5–500 milliseconds	Laser irradiation was started the moment the compression cell touched the bracket	Ceramic brackets (Transcendend series 6000 3M/UNITEK Monrovia, CA, USA) for laser debonding; 4-META MMA resin	No data	No data	2 W: - super pulse +1.4 °C 3 W: - super pulse +2.1 °C - normal pulse +2.7 °C
Iijima, M. [47]	CO_2_ laser: - wavelength: 10.6 μm - continuous wave - spot diameter: 0.45 mm - power outputs tested: 3 W, 4 W, 5 W, and 6 W - application time: 5 s per spot - distance from bracket: approximately 1 mm - applied to the 4 bracket wings (5 s each)	Immediate mechanical debonding with a universal testing machine (EZ Test, Shimadzu, Kyoto, Japan) using knife-edged shearing blade parallel to buccal surface; crosshead speed: 0.5 mm/min.	Brackets: single-crystal brackets (Inspire ice, Ormco, Orange, CA, USA) Adhesive: 1. Conventional etch and rinse adhesive (Transbond XT, 3M Unitek, Monrovia, CA, USA) 2. Self-etching adhesive (Transbond Plus, 3M Unitek, Monrovia, CA, USA)	Mean: 1. Conventional: control = 15.5 MPa 3 W, 10 MPa 4 W, 10 MPa 5 W, 10 MPa 6 W, 8 MPa 2. Self-etching: control = 12 MPa 3 W, 9 MPa 4 W, 5 MPa 5 W, 4 MPa 6 W, 3 MPa	1. Conventional: Control: Score 1 × 4 Score 2 × 1 6 W: Score 1 × 4 Score 2 × 1 2. Self-etching: Control: Score 0 × 1 Score 1 × 4 3 W: Score 1 × 5 4 W: Score 0 × 1 Score 1 × 4 5 W: Score 1 × 5 6 W: Score 1 × 5	Low output (3 W and 4 W): increase of about 100 °C to 150 °C High output (5 W and 6 W): increase of 200 °C Temperature returned to room temperature within 30 s after irradiation for all power settings
Mimura, H. [48]	CO_2_ laser: - wavelength: 10.6 μm - power outputs: 3 W and 7 W - tip placement: just apart from bracket - continuous application	Mechanical removal using shear force with force applied perpendicular to bracket–enamel interface (initial force 3 kgf, 1 mm/min speed).	Brackets: polycrystalline alumina brackets (Transcend series 6000, Unitek/3M, Monrovia, CA, USA) Adhesive: 1. Bis-GMA composite resin (Concise, 3M, Monrovia, CA, USA) 77% quartz filler 2. 4-META MMA resin (Super-Bond), no filler	Mean: 1. Concise groups: control = 14.81 kgf 3 W = 5.85 kgf 7 W = 4.01 kgf 2. Super-Bond groups: control = 12.49 kgf 3 W = 3.63 kgf 7 W = 3.41 kgf	MARI 1. Concise group: Control: Score 1 × 0 Score 2 × 3 Score 3 × 5 Score 4 × 12 3 W laser: Score 1 × 2 Score 2 × 6 Score 3 × 7 Score 4 × 5 7 W laser: Score 1 × 0 Score 2 × 6 Score 3 × 9 Score 4 × 5 2. Super-Bond: Control: Score 1 × 0 Score 2 × 2 Score 3 × 6 Score 4 × 12 3 W laser: Score 1 × 0 Score 2 × 1 Score 3 × 6 Score 4 × 13 7 W laser: Score 1 × 0 Score 2 × 1 Score 3 × 3 Score 4 × 16	No measurements during the deboning process. Thermal expansion: - Super-Bond: expanded until 80 °C (peak at 60 °C), then began contracting above 80 °C - Concise: showed linear expansion with temperature increase (4× greater than bracket expansion)
Tehranchi, A. [49]	CO_2_ laser: - power density: 50 W - exposure time: 5 s - pulse duration: 500 μs - interval between pulses: 2000 μs - frequency: 400 Hz - spot size: 1 mm - application: at center of brackets	Mechanical removal using the Instron machine blade immediately after the laser stopped with constant speed of 1 mm/min.	Brackets: polycrystalline alumina brackets (G & H Series, Schönheide, Germany) positioned 4 mm from incisal edge Adhesive: chemically cured orthodontic composite resin (No-mix, Unitek, Blue Bell, PA, USA)	Means: Control group: 23.7607 Laser group: 9.9145	ARI (Kruskal–Wallis test) Control: - mean rank: 11.53 - debonding site closer to enamel–adhesive interface Laser: - mean rank: 30.63 - debonding site closer to bracket surface ARI (U test) Control: mean 8.93 Laser: mean 22.07	No measurements during the procedure.
Demirkan [50]	Tm:fiber laser 1940 nm Time: 7 or 10 s Energy: 21, 25, or 30 J Power: 2.5 or 3 W Beam diameter: 400 μm	Brackets were irradiated with a scanning or non-scanning method. SBS was measured during irradiation.	Polycrystalline brackets (GH. Franklin, IN, USA) Adhesive: 3M, Unite Bonding Adhesive Set, Monrovia, CA, USA	Lack of precise date	No data	Scanning method: 2.5 W, 7 s = 5.02 (1.67) °C 3.0 W, 7 s = 3.56 (0.92) °C 2.5 W 10 s = 4.27 (0.89) °C 3 W 10 s = 6.21 (3.45) °C Non-scanning method: 2.5 W, 7 s = 3.86 (1.20) °C 3.0 W, 7 s = 4.82 (3.10) °C 2.5 W, 10 s = 5.57 (2.06) °C 3.0 W, 10 s = 3.92 (0.89) °C
Dostalova [51]	Tm:YAP laser 1998 nm Time: 60 s Power: 1 or 4 W Beam diameter: 3 mm	Brackets were debonded with pliers after irradiation.	Brackets: Fascination 2 (Dentaurum, Ispiringen, Germany) or Charity SL APC (3M Unitek Orthodontic Products, Monrovia, CA, USA) Adhesive: ConTec LC (Dentaurum, Ispringen, Germany).	No data	No data	Mean increase Fascination 1 W = 0.9 (0.5) °C 4 W = 2.8 (0.9) °C Charity 1 W = 0.7.(0.3) °C 4 W = 2.6 (1.1) °C
Tatjana Dostalova [52]	Tm:YAP Laser Irradiation 1998 nm Time: 60 s Power: 1 and 2 W Beam diameter: 3 mm Fluence: 849 or 1698 J/cm^2^ Irradiance: 14 or 28 W/cm^2^ Water flow: 2 mL/min. Spot size: 3 mm	After a period of 60 sec, the ceramic bracket was removed from the tooth surface mechanically, with 3M Unitek band-removing pliers (Unitek, Monrovia, CA, USA).	Fascination 2 (Dentaurum, Pforzheim, Germany) + Adhesive: ConTec LC adhesive (Dentaurum, Ispringen, Germany) BIS-GMA Charity SL APC (3M Unitek Orthodontic Products, Monrovia, CA, USA) + Adhesive: Transbond plus (3M Unitek Orthodontic Products, Monrovia, CA, USA) (Bis- GMA/TEGDMA (triethylene glycol dimethacrylate-based SEP adhesive system) self-etching primer	No data	No data	1 W: - Fascination 2 +3 °C - Charity +3.8 °C
Dostálová, T. [53]	1. GaAlAs diode laser: - wavelength: 808 nm - maximum output power: 20 W - fiber core diameter: 400 μm - numerical aperture: 0.22 - 1–10 W power settings - time of irradiation: 60 s 2. Tm:YAP laser: - wavelength: 1980 nm - maximum output power: 3.8 W - 1–2 W power settings - time of irradiation: 30, 60, or 90 s	After irradiation, brackets were removed mechanically.	Brackets: ceramic brackets Fascination 2 (Dentaurum, Ispringen, Germany) Adhesive: ConTec LC (Dentaurum, Ispringen, Germany) Primer: ConTec Primer Etching: ConTec Etch (applied for 15 s)	No data	No data	1. GaAlAs: without cooling - 1 W, 60 s: 18 °C increase - 2 W, 60 s: 29 °C increase - 10 W, 60 s: 114 °C increase No successful debonding 2. Tm:YAP: Without cooling: - 1 W, 60 s: 31 °C increase With cooling: - 1 W, 60 s: 2 °C increase - 1 W, 90 s: 5 °C increase - 2 W, 60 s: 9 °C increase Temperature monitored using NiCr-Ni thermocouple and thermal imaging camera
Xianglong Han [54]	Nd:YAG laser 1060 nm Time: 3 s Distance: 1 mm Power: 3 W Beam diameter: 0.6 mm Pulse width: 0.2 ms	Brackets were removed with shear debonding force. Laser was also used in 3 groups.	- metallic (MBT, 3M Unitek, Monrovia, CA, USA) and -polycrystalline ceramic brackets (Clarity, 3M Unitek,Monrovia, CA, USA) - orthodontic adhesive following the manufacturers’ recommendations.	Group: 1. Metallic brackets 9.78 MPa 2. Ceramic brackets 11.63 MPa 3. Ceramic brackets + laser irradiation 5.13 MPa	Group 1 Score 1 × 1 Score 2 × 2 Score 3 × 2 Score 4 × 3 Score 5 × 2 Score 6 × 0 Group 2 Score 1 × 1 Score 2 × 0 Score 3 × 1 Score 4 × 2 Score 5 × 4 Score 6 × 2 Group 3 Score 1 × 3 Score 2 × 4 Score 3 × 2 Score 4 × 0 Score 5 × 0 Score 6 × 1	No data
Hayakawa, K. [55]	Nd:YAG laser: - wavelength: 1060 nm - maximum output: 1.2 ms - pulse duration: 3.0 J with 5 pulses per second - single pulse per location (1 pps) - energy levels tested: 1.0 J, 2.0 J, 3.0 J - fiber waveguide diameter: 0.4 mm - output energy from fiber tip was 8.9% lower than nominal output power - tip distance from bracket: 0.1 mm without direct contact - applied to 2 spots: mesiodistal center of gingival surface and coronal surface under each bracket wing	1. Immediate removal (2.0 J and 3.0 J groups): no mechanical force needed; laser application caused spontaneous debonding. 2. Non-immediate removal: universal testing machine was used for brackets that did not debond spontaneously.	Brackets: 1. Single crystal ceramic brackets (Inspire, Shofu, Kyoto, Japan) 2. Polycrystalline ceramic brackets (Clarity, 3M Unitek, Monrovia, CA, USA) Adhesive: 1. 4-META/MMA based adhesive without fillers (Super-Bond, Sun Medical, Moriyama, Japan) 2. Bis-GMA-based photoactivated adhesive with fillers (Transbond, 3M Unitek, Monrovia, CA, USA)	Mean: Control = 25–30 MPa 1.0 J = 20–25 MPa 2.0 J = 10–15 MPa 3.0 J = 5–10 MPa	No data	Maximum temperature rise: 5.1 °C. Mean temperature increases by group: Single crystal + MMA: 2.0 J (1.71 °C), 3.0 J (2.46 °C) Single crystal + Bis-GMA: 2.0 J (1.74 °C), 3.0 J (1.67 °C) Polycrystalline + MMA: 2.0 J (1.09 °C), 3.0 J (1.44 °C) Polycrystalline + Bis-GMA: 2.0 J (1.07 °C), 3.0 J (2.08 °C) Temperature peak occurred at 0.5 s after irradiation and returned to baseline after 3 s.
Downarowicz [56]	Er,Cr:YSGG laser 2780 nm Time: 5–25 s Distance: 1–2 mm Power: 2.78–2.85 W Energy: 185–190 mJ Beam diameter: 0.6 mm Frequency: 25 Hz Pulse duration: 300 μs Er:YAG laser 2940 nm Time: 5–15 s Distance: 1–2 mm Power: 4 W Energy: 200 mJ, Beam diameter: 0.8 mm Frequency: 20 Hz Pulse duration: 300 μs	The brackets were irradiated by a laser until spontaneous debonding occurred.	Brackets: Inspire-ICE (Ormco, Glendora, CA, USA) Adhesive: Transbond XT (3M Unitek, Maplewood, MN, USA)	No data	No data	Er,Cr:YSGG Outside: 23.3 °C Inner: 21.4 °C Er:YAG Outside: 24.7 °C Inner: 24.2 °C
Yilanci [57]	Er:YAG laser 2940 nm Time: 4–6 s Power: 1.2 W Energy: 600 mJ Beam diameter: 1.3 mm Surface area: 0.004225 cm^2^ Frequency: 2 Hz Power density: 90.4 W/cm^2^ Fluence: 45.2 J/cm^2^	Brackets were removed with help of laser light after thermocycling (group B) or without thermocycling (group A).	Monocrystalline brackets (Radiance, American Orthodontics, Sheboygan, WI, USA) Adhesive: Transbond XT (3 M; Unitek, Monrovia, CA, USA)	No data	No data	Mean change Group A Incisors = +2.12 °C Premolars = +2.26 °C Group B Incisors = +2.61 °C Premolars = +1.74 °C
Mocuta [58]	Er:YAG laser 2940 nm Distance: 1 mm Energy: 600 mJ Pulse duration: 800 μs Beam diameter: 1.3 mm Frequency: 2 Hz	Brackets were debonded using Er:YAG laser-assisted action.	Monocrystalline brackets Adhesive: no data	No data	No data	No data
Dostalova [59]	Er:YAG 2 940 nm Time: 140 s Peak power: 1 kW Energy: 280 mJ Beam diameter: 1 mm Frequency: 6 Hz Power density: 144 kW/cm^2^ Fluence: 36 J/cm^2^	The locks were irradiated for 140 s, then debonded using special pliers.	Brackets: Clarity Advanced (3M Unitek, Monrovia, CA, USA) Adhesive: Transbond XT (3M Unitek, Monrovia, CA, USA) or Variolink II Professional Set (Ivoclar Vivadent AG, Schaan, Liechtenstein)	No data	No data	Increase from 2.2 °C to 3.0 °C
Mirhashemi [60]	Er,Cr:YSGG laser Time: 10 s Distance: 2 mm Power: 3 W Beam diameter: 800 μm Fluence: 22/28 J/cm^2^ Er: YAG laser Time: 10 s Pulse duration: 100 μs Distance: 2 mm Power: 3 W Beam diameter: 1 mm Frequency: 20 Hz Fluence: 22/28 J/cm^2^	SBS was measured immediately after laser irradiation to three sides of the bracket bases.	Brackets: GAC International Inc. (Islandia, NY, USA) Adhesive: Transbond XT (3M Unitek, Monrovia, CA, USA).	Mean Er,Cr:YSGG laser = 18.03 MPa Er:YAG laser = 17.01 MPa	Er,Cr:YSGG laser Score 0 × 1 Score 1 × 9 Score 3 × 2 Er:YAG laser Score 1 × 8 Score 2 × 2 Score 3 × 2	No data
Mundethu [61]	Er:YAG 2940 nm Time: one pulse Distance: in contact Energy: 600 mJ Pulse duration: 800 μs Beam diameter: 1.3 mm Frequency: 2 Hz	Debonding was performed using a laser tip in contact with the center of the bracket.	Polycrystalline brackets (Damon Clear; Ormco Corp, Orange, CA, USA) Adhesive: Blugloo (Ormco Corp, Orange, CA, USA)	No data	Score 3 for all specimens	No data
Tozlu [62]	Er:YAG laser 2940 nm Time: 6 s Distance: 2 mm Power: 5 W Beam diameter: 1 mm	Debonding with SBS measurement was performed 1 s, 18 s, 30 s, or 60 s after laser exposure.	Polycrystalline brackets (Transcend series 6000, 3M Unitek, Monrovia, CA, USA) Adhesive: Transbond XT (3 M Unitek, Monrovia, CA, USA)	1 s = 2.74 ± 1.99 MPa 18 s = 10.36 ± 2.12 MPa 30 s = 16.38 ± 2.25 MPa 60 s = 18.11 ± 2.40 MPa	1 s Score 1 × 1 Score 2 × 6 Score 3 × 13 18 s Score 1 × 3 Score 2 × 7 Score 3 × 10 30 s Score 1 × 3 Score 2 × 9 Score 3 × 8 60 s Score 1 × 4 Score 2 × 10 Score 3 × 6	No data
Hoteit [63]	Er,Cr:YSGG laser (Waterlase MD, Biolase technology, Inc., Irvine, CA, USA) Wavelength: 2780 nm MX7 sapphire tip Beam diameter: 0.7 mm at the impact point Time: 6 s 70% air and 30% water Er:YAG laser (Fidelis; Fotona, Medical laser, Ljubljana, Slovenia) Wavelength: 2940 nm using 0.9 mm as a beam diameter at the impact point.	Six groups were debonded using Er,Cr:YSGG. Eight groups were debonded with an Er:YAG laser.	Brackets: adhesive pre-coated bracket (APC) Flash-free, 3M clarity advance ceramic brackets, Monrovia, CA, USA Adhesive: Transbond XT bonding (3M Unitek, Monrovia, CA, USA) 15 experimental groups based on various Er:YAG settings	The mean shear bond strength (SBS) levels: Er,Cr:YSGG 5 W/20 Hz: 5.30 ± 5.26 MPa Control group: 21.07 ± 1.80 MPa	No data	No data
Hamadah [64]	Er:YAG laser (Lightwalker^®^ ST-E, 8 W, Fotona Inc., Ljubljana, Slovenia) Wavelength: 2940 nm Distance: 0.7 cm Laser spot size: 0.9 mm Pulse duration: 50, 100, and 300 μs Frequency: 30 Hz Water/air: 2 mL/s and 2 mL/s	Brackets were exposed to the Er:YAG laser for 6 s using the laser-scanning method.	Brackets: ceramic brackets (20/40™ Ceramic Brackets, American Orthodontics, Sheboygan, WI, USA) Adhesive: orthodontic composite (3M Unitek^®^, Transbond^®^ XT, Monrovia, CA, USA)	No data	Group 1: 2, 3 Group 2: 3 Group 3: 3	No data
Nalbantgil [65]	Er:YAG laser (VersaWave, Hoya ConBio, Fremont, CA, USA) Wavelength: 2940 nm Pulse repetition rate: 20 Hz Pulse duration: 300 ms Water spray: 40–50 mL/min Tip Diameter: 1 mm Laser irradiation for the three study groups: 1. 2 W(100 mJ at 20 Hz) 2. 4 W (200 mJ at 20 Hz) 3. 6 W (300 mJ at 20 Hz)	To assess the debonding site, adhesive remnant index (ARI) scores were noted. A thermocouple was used to prepare 60 human premolar teeth at the same energy levels and in the same manner in order to measure intrapulpal thermal increase.	Brackets: polycrystalline alumina brackets (Transcend series 6000; 3M Unitek, Monrovia, CA, USA) Adhesive: Transbond XT (3M Unitek, Monrovia, CA, USA) orthodontic adhesive system	No data	There was no statistical difference among experimental groups, excluding the control group.	The temperature increases were Group 2 W: 0.67 ± 12 °C Group 4 W:1.25 ± 0.16 °C Group 6 W: 2.36 ± 0.23 °C
Didem Nalbantgil [66]	ER:YAG laser 2940 nm Time: 3, 6, 9 s Distance: 2 mm Power: 4.2 W Beam diameter: 1 mm Frequency: 30 Hz Energy: 140 mJ	Debonding with shear test was carried out 45 s after the laser pulse; laser irradiation lasted 3, 6, 9 s. Control group without irradiation.	Polycrystalline alumina incisor brackets (Transcend series 6000, 3M Unitek, Monrovia, CA, USA) Adhesive: Transbond XT (3M Unitek, Monrovia, CA, USA) and light-cured with halogen light curing unit (Optilux, Kerr, Orange, CA, USA)	Control group: 22.76 MPa 3 s irradiation: 12.38 MPa 6 s irradiation: 10.75 MPa 9 s irradiation: 8.81 MPa	Control group: Score 0 × 1 Score 1 × 5 Score 2 × 10 Score 3 × 4 3 s group: Score 0 × 0 Score 1 × 3 Score 2 × 8 Score 3 × 9 6 s group: Score 0 × 0 Score 1 × 5 Score 2 × 6 Score 3 × 9 9 s group: Score 0 × 0 Score 1 × 0 Score 2 × 5 Score 3 × 15	3 s group: +1.27 °C 6 s group: +2.79 °C 9 s group: + 4.59 °C
Oztoprak, M. O. [67]	Er:YAG laser: wavelength: 2940 nm - power: 4.2 W - duration: 9 s per bracket - application method: scanning motion horizontally parallel to bracket slot - tip distance from bracket: 2 mm	Mechanical removal 45 s after laser exposure using shear force. Force applied occluso-gingivally using a universal testing machine.	Brackets: polycrystalline ceramic brackets (Transcend series 6000, 3M Unitek, Monrovia, CA, USA) Adhesive: Transbond XT (3M Unitek, Monrovia, CA, USA)), light-cured for 40 s	Mean: Control group = 20.75 MPa Laser group = 9.52 MPa	Control group: Score 0 × 3 Score 1 × 11 Score 2 × 12 Score 3 × 4 Laser group: Score 1 × 1 Score 2 × 10 Score 3 × 19	No measurements during the procedure.
Alakuş-Sabuncuoǧlu, F. [68]	Er:YAG laser - wavelength: 2940 nm - mode: maxi short pulse (MSP) - pulse width: 100 μs - pulse frequency: 10 Hz - power: 3 W - energy per pulse: 120 mJ - duration: 6 s - application method: scanning in reverse S pattern - tip distance: 1 mm from bracket - used with air and water cooling	Mechanical removal using universal testing machine (Shimadzu Autograph AG-IS) with speed: 0.5 mm/min.	Brackets: polycrystalline ceramic brackets (Transcend series 6000, 3M Unitek, Monrovia, CA, USA) Adhesive: Transbond XT (3M Unitek, Monrovia, CA, USA)), light-cured for 10 s from 4 sides (total 40 s)	Mean: Control group: 13.42 ± 1.23 MPa Laser group: 8.47 ± 0.71 MPa	Control group: Score 0 × 2 Score 1 × 4 Score 2 × 4 Score 3 × 0 Laser group: Score 0 × 0 Score 1 × 1 Score 2 × 4 Score 3 × 5	No measurements during the procedure.
Rao [69]	Er,Cr:YSGG laser 2780 nm Distance: 1 mm Power: 4.5 or 6 W	Following laser irradiation, the brackets were removed as per the manufacturer’s instructions.	Brackets: no data Adhesive: Transbond XT (3 M Unitek, Monrovia, CA, USA)	No data	Mean: 4.5 W = 1.33 (0.62) 6 W = 1.07 (0.59)	No data
Abdulaziz [70]	Er,Cr:YSGG laser (Waterlase iPlus; Biolase Technology Inc., Irvine, CA, USA) Wavelength: 2780 nm Frequency: 20 Hz Power: 4 W Tip diameter: 0.6 mm Pulse duration: 60 μs Level of air/fluid cooling: 70% air and 30% Time: 6 s	Scanning electron microscopy was used for the evaluation of the enamel’s microstructure, surface roughness following polishing, intrapulpal temperature increase, and adhesive remnant index (ARI).	Brackets: monocrystalline ceramic brackets (Perfect Clear, Hubit, Uiwang-si, Republic of Korea) Adhesive: Adhesive bond following Orthosolo (ORMCO, Orange, CA, USA). Debonding: Er,Cr:YSGG laser applications.	No data	Compared to the circular group, the conventional group had a substantially greater percentage of adhesive remnant index values of 2 and 3. Compared to the scanning group, the traditional group had a noticeably greater percentage of adhesive remnant index values of 2 and 3.	There was a significantly higher average intrapulpal temperature increase in the circular group (1.9 ± 0.5 °C) compared to the scanning group (0.9 ± 0.2 °C)
Stein [71]	445 nm diode laser Time: 15 s Distance: in contact Beam diameter: 320 μm CW Power density: 2 W/cm^2^ Fluence: 30 J/cm^2^	Debonding was performed using the laser tip in contact mode at a 90° angle to the bracket surface.	Polycrystalline brackets In-Ovation C (GAC, Grafelfing, Germany). Adhesive: Transbond XT (3M/Unitek, Monrovia, CA, USA).	No data	No data	Mean: Inner = 38.15 (0.51) °C Outside = 39.58 (2.38) °C
Stein [72]	445 nm diode laser Time: 15 s Power: 2 W Beam diameter: 320 μm CW	Immediately after laser application, the bracket was removed with bracket-removal pliers.	Polycrystalline brackets In-Ovation C (GAC, Grafelfing, Germany). Adhesive: Transbond XT (3M/Unitek, Monrovia, CA, USA).	No data	Eye/10-fold magnification: Score 0 × 9 Score 2 × 2 Score 3 × 4 20-fold magnification: Score 0 × 8 Score 1 × 1 Score 2 × 4 Score 3 × 2	No data
Stein [73]	445 nm diode laser Time: 15 s Distance: in contact Beam diameter: 320 μm CW Power density: 2 W/cm^2^ Fluence: 30 J/cm^2^	SBS was measured immediately after laser irradiation to three sides of the bracket bases.	Polycrystalline brackets In-Ovation C (GAC, Grafelfing, Germany) Adhesive: Transbond XT (3 M Unitek, Monrovia, CA, USA)	Mean = 10.08 MPa	Score 1 × 7 Score 2 × 7 Score 3 × 1	No data
Yassaei [74]	Diode laser (Fox, ARC, Nürnberg, Germany) Wavelength: 980 nm Power: 2.5 W Time: 10 s Distance: 5 mm	The adhesive remnant index (ARI), lengths, and frequency of enamel cracks were examined between the groups following debonding. It was also measured how much the intrapulpal temperature increased.	Brackets: polycrystalline ceramic brackets (Allure, Whitinsville, MA, USA Adhesive: bonding primer (Resilience, Ortho Technology, Tampa, FL, USA)	No data	Conventional debonding Group 1: 1 (6.7%) Group 2: 3 (20%) Group 3: 8 (53.3%) Group 4: 3 (20%) Laser debonding Group 1: 1 (6.7%) Group 2: 7 (46.7%) Group 3: 5 (33.3%) Group 4: 2 (13.3%)	Changes in temp: 1.46 °C
Feldon [75]	Diode laser 2 or 5 W/cm^2^	At two laser energy levels—2 and 5 W per square centimeter—the shear bond strength and heat effects on the pulp chamber were evaluated. Significant variations in shear bond strength values were identified.	Brackets: Inspire ICE (Ormco, Orange, CA, USA), a monocrystalline bracket; and Clarity (3M Unitek, Monrovia, CA, USA), a polycrystalline bracket Adhesive: single-paste visible light-cured orthodontic adhesive system, Transbond XT (3M Unitek, Monrovia, CA, USA) Both monocrystalline and polycrystalline ceramic brackets were attached to the maxillary central incisors of cows	Mean shear bond strength Group 1: 9.79 MPa Group 2: 9.68 MPa Group 3: 7.24 MPa Group 4: 15.99 MPa Group 5: 9.27 MPa Group 6: 8.88 MPa	There were no significant ARI score differences between any of the groups tested. Uniformly, they all had a mean ARI score of or close to 3.	The mean increases in pulp chamber temperature or groups 3, 4, and 5 were statistically significantly less than the 5.5 °C increase threshold and not significantly different from the 1.8 °C standard. Group 6 had a mean pulp chamber increase significantly greater than the 1.8 °C standard and not significantly different from the 5.5 °C standard.
Ayşe Sena Kabaş Sarp [76]	Ytterbium fiber laser 1070 nm Distance: 15 cm Power: 20 W Beam diameter: 1.6 mm Laser mode: CW and modulated mode	The laser was turned on synchronously with shear load application and turned off when the bracket was debonded.	Polycrystalline ceramic brackets (G&H, Franklin, IN, USA) Adhesive: chemically curing Bis-GMA resin (3M, Unite Bonding Adhesive Set, St. Paul, MN, USA)	No Data	No Data	CW mode: 1. Control group 2. +1.77 °C 3. +3.2 °C 4. +3.7 °C 5. +8.6 °C Modulated mode: 200/600 + 2.7 °C 300/900 + 3.1 °C 400/1200 + 2.9 °C

**Table 2:** Quality assessment.

Author	Sample Size Calculation	Group Size of at Least 10 Subjects	Control Group	Detailed Description of Laser Parameters	Detailed Description of the Debonding Method	Randomization	Total	Risk of Bias
Khalil [3]	1	1	1	1	1	1	6	Low
Tocchio [12]	0	1	0	1	1	1	4	Moderate
Grzech-Leśniak [13]	0	1	1	1	1	0	4	Moderate
Arima [39]	0	1	1	1	1	1	5	Low
Macri [40]	0	1	1	1	1	1	5	Low
Ahrari [41]	0	1	1	1	1	1	5	Low
Matos [42]	0	1	1	0	1	1	4	Moderate
Saito [43]	0	1	0	1	1	1	4	Moderate
Strobl [44]	0	1	0	0	1	0	2	High
Ma [45]	0	1	1	1	1	0	4	Moderate
Obata [46]	0	1	1	0	0	0	2	High
Iijima [47]	0	1	1	1	1	1	5	Low
Mimura [48]	0	1	1	0	1	0	3	Moderate
Tehranchi [49]	0	1	1	1	1	0	4	Moderate
Demirkan [50]	0	0	1	1	1	0	3	Moderate
Dostalová [51]	0	1	1	0	1	0	3	Moderate
Dostalova [52]	0	1	1	1	1	0	4	Moderate
Dostálová [53]	0	1	0	1	1	0	3	Moderate
Han [54]	0	1	0	1	0	0	2	High
Hayakawa [55]	0	1	1	1	1	1	5	Low
Downarowicz [56]	0	1	0	1	1	0	3	Moderate
Yilanci [57]	0	1	0	1	0	0	2	High
Mocuta [58]	1	1	0	1	0	1	4	Moderate
Dostalova [59]	0	1	1	1	1	0	4	Moderate
Mirhashemi [60]	0	1	1	1	1	0	4	Moderate
Mundethu [61]	0	1	0	1	0	0	2	High
Tozlu [62]	0	1	1	1	1	1	5	Low
Hoteit [63]	0	1	1	1	0	1	4	Moderate
Hamadah [64]	0	1	0	1	0	1	3	Moderate
Nalbantgil [65]	0	1	1	1	0	1	4	Moderate
Nalbantgil [66]	0	1	1	1	1	1	5	Low
Oztoprak [67]	0	1	1	1	1	1	5	Low
Alakuş-sabuncuoğlu [68]	0	1	1	1	0	1	4	Moderate
Rao [69]	0	1	1	0	1	1	4	Moderate
Abdulaziz [70]	1	1	1	1	1	0	5	Low
Stein [71]	1	1	0	1	1	0	4	Moderate
Stein [72]	1	1	0	1	1	1	5	Low
Stein [73]	0	1	1	1	1	1	5	Low
Yassaei [74]	0	1	0	1	1	1	4	Moderate
Feldon [75]	0	1	1	0	1	0	3	Moderate
Sarp [76]	0	0	1	1	1	0	3	Moderate

## Data Availability

Data supporting the findings of this study are available within the article.

## Supplementary Materials

The following supporting information can be downloaded at: https://www.mdpi.com/article/10.3390/jfb16040123/s1, Table S1. General characteristics of studies.
