# Supplementary material for: Orthodontic Ceramic Bracket Removal Using Lasers: A Systematic Review
Source: J Funct Biomater. 2025 Apr 1;16(4):123. doi: 10.3390/jfb16040123 (PMC12027597; doi:10.3390/jfb16040123)
Supplement: Supplementary file 1 [file jfb-16-00123-s001.zip › jfb-3507506-supplementary.pdf]

**Table S1.** General characteristics of studies

| Study | Aim of the study | Material and Methods | Results | Conclusions |
|---|---|---|---|---|
| Khalil [3] | This study set out to assess and contrast several methods for debonding ceramic brackets in terms of adhesive residual index and shear bond strength. | One hundred removed premolars were divided into five groups at random. Composite resin that had been light-cured was then used to attach ceramic bracklets to teeth. Group I was used as a control, Group II was used for chemically assisted debonding using peppermint oil, Group III was used for ultrasonic debonding, Group IV was used for diode laser assisted debonding, and Group V was used for Er:YAG laser assisted debonding. Shear testing of the brackets was done with a universal testing machine, and then ARI assessment and scanning electron microscopy were used to evaluate the enamel microstructure. | The ultrasonic, diode, and Er:YAG laser groups showed a noticeably decreased shear bond strength. The chemical group did not differ significantly. Only the Er:YAG laser group showed a noticeably greater adhesive residual index with little changes to the enamel microstructure. | A promising technique for debonding ceramic brackets is the Er:YAG laser. Shear bond strength was considerably decreased by diode laser and ultrasonic. However, there was no difference in the adhesive remnant index between the two groups. Without more research, chemically assisted debonding cannot be advised due to its limited effectiveness. |
| Tocchio [12] | Through thermal softening, thermal ablation, or photoablation, laser light energy has been demonstrated in various investigations to break down resins. Both bracket and enamel fracture during debonding could be avoided if this method is effectively used for bracket debonding. | Using the acid-etch method typically used in dentistry, orthodontic brackets made of polycrystalline alumina and single crystal alumina (sapphire) ceramic were attached to the labial surfaces of lower deciduous bovine incisor teeth. The brackets were debonded by exposing their labial surfaces to XeCI excimer laser light with wavelengths of 248 nm, 308 nm, and 1060 nm with light power densities ranging from around 3 to 33 W/cm 2 under an externally induced stress of either 0.8 MPa or zero MPa. To ascertain the degree of bracket and enamel damage, debonding periods were recorded and the surfaces produced by debonding were inspected using light and scanning electron microscopy. | The findings demonstrated that no sample had any enamel or bracket degradation under the study's circumstances. For radiation at 248 nm, 308 nm, and 1060 nm, the polycrystalline brackets debonded in roughly 3 seconds, 5 seconds, and 24 seconds, respectively. When the bracket is heated, the bonding glue becomes softer, which leads to the debonding of polycrystalline brackets. The tooth is removed by sliding the heated bracket ttien off. Within a second, every sapphire bracket debonded. | This unique debonding process merits more research given the growing use of lasers in dentistry and the sharp decline in the cost of these instruments. Laser dcbonding offers the practitioner a very quick and painless debonding process without the possibility of bracket fractures or enamel tearouts. |

| | | | | |
|---|---|---|---|---|
| Grzech-Leśniak [13] | To assess how the pulp temperature and enamel surface are affected when brackets are removed with an erbium laser. | For orthodontic indications, 55 caries-free premolars were extracted and a total of 55 brackets (20 metal and 35 ceramic) were bonded to them. The Er:YAG laser was used to irradiate the brackets. | When compared to the circular motion technique around the metal or ceramic brackets, the scanning method has resulted in a noticeably reduced temperature increase. The study groups' ARI scores did not differ from one another. After laser-assisted debonding, SEM analysis showed no enamel surface cracks, in contrast to the control samples that had cracks. When compared to control samples, EDS revealed a greater mean percentage of calcium in all test groups. Compared to ceramic brackets, metal brackets have a higher concentration of calcium components. | When Er:YAG laser-assisted debonding is used instead of traditional bracket removal, the pulp temperature is slightly raised and there is a lower chance of enamel damage. |
| Arima [39] | Using light-cured Bis-GMA resin containing heat-expandable microcapsules and a $CO_2$ laser as a method for removing ceramic brackets. | 60 bovine and 5 human teeth with bonded ceramic brackets bonded in various resin proportions were treated with a $CO_2$ laser. The temperature at the base of the bracket, SBS and ARI were measured on the bovine teeth, and the intrachamber temperature on the human teeth. | When using 25% of the tested resin after laser irradiation, SBS is significantly reduced. The maximum increase in temperature in the pulp chamber was 5.3 degrees. | The use of an adhesive material containing 25% microcapsules provides sufficient bonding strength for orthodontic treatment and allows for quick and safe removal of brackets using a $CO_2$ laser. |
| Macri [40] | To investigate the temperature in the adhesive material and pulp chamber, SBS and ARI during debonding of ceramic brackets using a $CO_2$ laser. | Ceramic brackets were removed from 105 human teeth using a $CO_2$ laser. The temperature inside the chamber and at the base of the bracket was measured on 30 of them, while the remaining teeth were used to measure SBS and assess ARI. | When using 5W, 8W and 10W power and 0.01s pulse duration, the temperature increase was safe for the pulp. The greatest decrease in SBS was observed with the parameters 10W, 3s, 0.01s. The least composite residue (ARI=1.66) was achieved with the parameters 8W, 3s, 0.01s. | The use of a $CO_2$ laser reduces the SBS value without excessive temperature increase. |

| | | | | |
|---|---|---|---|---|
| Ahrari [41] | Evaluation of tooth enamel surface properties after removal of ceramic brackets with or without the use of laser light. | Ceramic brackets placed on human teeth were treated with a $CO_2$ laser using different parameters while measuring the temperature inside the chamber. Then, ARI and damage to the enamel surface were assessed. | During laser debonding, the temperature increased within the safe range, no enamel damage was observed, and the ARI was reduced compared to the control group. | Laser debonding allows you to remove brackets without damaging the enamel and tooth pulp. |
| Matos [42] | Evaluation of SBS, ARI and debonding mechanism of ceramic brackets using a $CO_2$ laser. | Ceramic brackets were placed on human teeth using different adhesive materials. After $CO_2$ laser irradiation, SBS and ARI were examined. | Laser treated brackets showed lower SBS, especially with chemical type of retention cemented with Z250. Z250 cemented brackets showed the highest ARI value. | $CO_2$ laser exposure facilitates the debonding of ceramic brackets by reducing the SBS value. |
| Saito [43] | The study's purpose was to investigate how a bracket bonded with an orthodontic glue that contained thermal expansion microcapsules and a $CO_2$ laser as the heating method may reduce the debonding strength and duration while still being safe. | Using bonding materials with different microcapsule contents (0, 30, and 40 weight percent), ceramic brackets were cemented to bovine permanent mandibular incisors. The bond strengths were assessed following laser irradiation for 4, 5, and 6 seconds, and compared to groups that were not exposed to laser light. | In comparison to the nonlaser groups, the bond containing 40 weight percent microcapsules showed a considerable decrease in bond strengths to approximately 0.40 to 0.48 times (4.6 to 5.5 MPa) after 5 or 6 seconds of laser irradiation. With laser irradiation for 6 seconds, the pulp chamber's mean temperature increased by 4.3 °C, which was less than what was needed to cause pulp damage. | Debonding ceramic brackets with minimal enamel damage or tooth pain may be possible with the safe and efficient use of a $CO_2$ laser in conjunction with an orthodontic glue that contains thermal expansion microcapsules. |
| Strobl [44] | To investigate efficiency of using $CO_2$ and Nd:YAG (neodymium: yttrium-aluminum-garnet) lasers in debonding ceramic brackets from the enamel surface. | The study used 93 human teeth with bonded ceramic brackets. 43 of them were treated with a $CO_2$ and Nd:YAG laser to assess the effect of this procedure on debonding the brackets. | Using a laser reduces the force required to remove the bracket, reduces the risk of enamel damage and lowers the ARI. | The use of $CO_2$ and Nd:YAG lasers to remove ceramic brackets makes the procedure easier and safer. |
| Tsun Ma [45] | Find a method to reduce the risk of ceramic bracket breakage during debonding with a | Bovine and human teeth with ceramic brackets were exposed to a $CO_2$ laser for 2 seconds. This is the time that causes a safe increase in temperature for the pulp (2 ° C). | Using a $CO_2$ laser before removing a ceramic bracket reduces the force required to remove the bracket, thereby | The study proves that the use of a $CO_2$ laser can increase the safety of debonding ceramic brackets |

| | | | | |
|---|---|---|---|---|
| | safe increase in pulp temperature. | | reducing the likelihood of breakage. | without damaging the pulp. |
| Akihito Obata [46] | The effect of the use of super pulse $CO_2$ laser on the bonding and debonding of orthodontic brackets. | A group of teeth were exposed to a $CO_2$ laser to test the effect of this procedure on the bonding of metal brackets. A group of teeth with ceramic brackets were also exposed to a $CO_2$ laser to assess the effect of this procedure on debonding. SBS, pulp temperature rise, and bracket debonding time were evaluated. | $CO_2$ laser etching resulted in lower SBS compared to chemical etching. The use of a $CO_2$ laser for debonding resulted in a shorter debonding time and an increase in pulp temperature to levels that are safe for its viability. | The study showed that the $CO_2$ laser was more helpful in the debonding process than it was in the bonding of the orthodontic brackets. |
| Iijima M. [47] | To assess if $CO_2$ laser used for debonding affects enamel's mechanical properties, mainly focusing on how strong and elastic the enamel remains after the procedure. | Human's 53 premolar teeth extracted from orthodontic patients were divided into 10 groups for different tests. The infrared thermographic microscope system was used for observing the teeth while applying different laser power (3-6W).<br>Bracket bond strength was measured using 2 different adhesives (conventional and self-etching).<br>Nanoindentation tests measured hardness and elasticity of the enamel.<br>Cross-sectional samples were used to monitor temperature changes. | $CO_2$ high laser power (5-6 W), increased the enamel's temperature to 200°C, while lower power (3-4 W) raised it to 100-150°C.<br>The strength of bracket bonding decreased after all laser treatments.<br>The hardness and elasticity of tooth enamel remained unchanged after laser bonding removal | Removal of ceramic brackets with a $CO_2$ laser appears to be safe as it has not caused the damage of the enamel's structure despite the temperature increases. |
| Mimura H. [48] | To investigate the effectiveness of laser removal of ceramic brackets with two different bonding materials. | The polycrystalline brakcets were attached to 123 maxillary premolars by Bis-GMA or 4-META MMA resin. Thereafter, $CO_2$ laser at different power settings (3 or 7 watts) was applied. Analysis included debonding force, removal time, energy needed for removal, tooth damage, remaining adhesive and thermal expansion of material. | The MMA resin performed significantly better than Bis-GMA. Moreover, MMA resin required less laser power, less total energy to remove brackets. MMA resin tended to stay on the tooth surface, while Bis-GMA came off with the bracket. Two adhesives have different thermal response. | $CO_2$ laser debonding proved to be an effective method, with MMA resin showing superior outcomes over Bis-GMA by requiring less power and preventing tooth damage. |
| Tehranchi A. [49] | To evaluate whether the $CO_2$ laser is an effective alternative to | 30 maxillary premolars divided into 2 groups. Laser group received super pulse $CO_2$ laser treatment. Several parameters were measured | The laser required less force to remove the brackets. Both methods caused the brackets to break away in similar | $CO_2$ laser proved superior to conventional methods for removing ceramic |

| | | | | |
|---|---|---|---|---|
| | traditional methods of brackets removal. | such as bond strength, debonding location, ARI. | locations. The conventional removal group showed less adhesive remaining on the teeth in comparison to the laser group. | brackets, providing gentler removal and better enamel protection. |
| Demirkan [50] | To investigate the optimal parameters of the 1940 nm Tm:fiber fiber laser for the removal of ceramic brackets. | Bovine teeth were used to debond ceramic brackets using a Tm:fiber laser, while the temperature change and the breakout force of the brackets were measured. | Effective debonding occurred at energies above 25J. Safe temperature increases were observed using the following parameters: 2.5-W 7-s nonscanning, 3.0-W 7-s scanning, and 3.0-W 10-s nonscanning irradiation method. | The use of a 1940 nm Tm:fiber laser may be a promising tool for debonding ceramic brackets. |
| Dostalova [51] | Investigation of the positive effect of laser radiation on the detachment of brackets. | Diode pumped Tm:YAP microchip lasers of 1W and 4W were used on ceramic brackets cemented to human teeth. Temperature was monitored. SEM images of the enamel surface were then taken. | The bond strength decreased at 1W, but increased at 4W. The temperature increase was safe for the pulp. | Laser radiation with a wavelength of 2 μm can be used to thermally soften adhesive materials. |
| Tatjana Dostalova [52] | Development of an efficient and tooth safe method for debonding ceramic brackets using the Tm:YAP laser | Two types of ceramic brackets were exposed to the Tm:YAP laser for debonding. The enamel structure, resin residue and pulp temperature increase were investigated. | The debonding of ceramic brackets with the Tm:YAP laser does not damage the enamel. The temperature increase was safe for the pulp in both cases. The irradiated brackets were removed with most of the adhesive. | The use of the Tm:YAP laser makes ceramic bracket debonding safer and more efficient than the traditional method. |
| Dostálová T. [53] | Finding the optimal laser-based method for removing ceramic brackets without damaging the tooth.. | Two different lasers (GaAlAs diode laser and Tm:YAP laser) were tested on 10 premolars extracted from orthodontic patients aged 11-15 years. Temperature changes were monitored with or without water cooling using thermocouples and thermal imager - infrared camera. Microscopy was used to assess enamel surfaces. Saline solution was used to store the teeth. | The GaAlAs laser (808 nm) proved ineffective for bracket removal notably heating the tooth rather than debonding the bracket. The Tm:YAP laser (1.98 μm) successfully debonded brackets (60 seconds, 1W power) with water cooling, causing minimal temperature increase (2°C). | The Tm:YAP laser with water cooling proved to be safe and effective, while the GaAlAs laser was not. |

| | | | | |
|---|---|---|---|---|
| | | | Without water cooling or with extended time exposure, both lasers caused unacceptable temperature rise. | |
| Xianglong Han [54] | Evaluation of the efficiency of Nd:YAG laser-assisted debonding of ceramic brackets | The study used 20 teeth with bonded ceramic brackets. Before debonding, 10 of them were treated with a Nd:YAG laser to assess the effect of this procedure on SBS and ARI. In addition, the enamel was examined by SEM after removal of the brackets. | The use of an Nd:YAG laser prior to debonding results in significantly lower SBS and ARI values.<br>The enamel of the teeth where the brackets were removed by laser remained intact, unlike the other teeth examined. | Debonding with an Nd;YAG laser is a more effective and safer technique for the patient. |
| Hayakawa K. [55] | To evaluate the effectiveness of a Nd:YAG laser as a method for safely removing ceramic orthodontic brackets from teeth. | The study tested Nd:YAG laser debonding of ceramic brackets (single and polycrystalline) on bovine mandibular incisors using two adhesives at three different laser energy levels. Bond strength, pulp temperature, and surface damage was measured to assess the effectiveness. | Single-crystal brackets came off more easily than polycrystalline ones at 2.0 joules, though this difference disappeared at 3.0 joules.<br>Both adhesive types performed similarly.<br>Tooth temperature stayed in safe limits. | Nd:YAG laser can be effectively and safely used to remove ceramic brackets. |
| Downarowicz [56] | Evaluation of pulp and enamel temperature changes during laser bracket removal. | Brackets were cemented onto 13 human teeth and debonded using Er:YAG and Er,Cr:YSGG lasers. Temperatures were measured inside the tooth chamber and at the base of the bracket. | The temperature difference between the enamel and the chamber was 1.4°C for the Er,Cr:YSGG laser and 0.6°C for the Er:YAG laser. | The use of both tested lasers for debonding of ceramic brackets is effective and safe. |
| Yilanci [57] | Evaluation of the temperature change in the pulp chamber during the use of Er:YAG laser for the removal of ceramic brackets. | An uncooled Er:YAG laser was used on 40 human teeth with cemented ceramic brackets and the temperature inside the tooth chambers was measured. | Intrapulpal temperature increased more in incisors than in premolars. A greater increase in temperature was observed with increasing exposure time. | The use of tested laser for debonding of ceramic brackets is effective and safe. |
| Mocuta [58] | Evaluation of the Er:YAG laser ceramic bracket removal technique compared to conventional methods. | The study was conducted on 10 patients, checking their subjective feelings, debonding time and microdynamics of blood flow in the pulp. | In the Wong–Baker FACES Pain Rating Scale, laser debonding received the maximum score of 2, while conventional debonding received a score of 6. | Bracket removal using Er:YAG laser radiation is faster and more comfortable for the patient than the |

| | | | | |
|---|---|---|---|---|
| | | | Conventional debonding takes significantly longer time. No significant difference in blood flow to the pulp was observed. | conventional procedure. |
| Dostalova [59] | Evaluation of the effect of Er:YAG laser radiation on the detachment of metal and ceramic brackets and damage to enamel | 10 ceramic brackets were bonded to human teeth and irradiated with an Er:YAG laser with temperature measurement during the procedure. After the brackets were removed, the surface was assessed by SEM. | Debonding of brackets was easier after irradiation. Temperature increased slightly. SEM showed no damage to enamel. | Exposing ceramic brackets to an Er:YAG laser makes them much easier to remove and reduces the amount of remaining adhesive. |
| Mirhashemi [60] | Evaluation of the effect of Er:YAG and Er,Cr:YSGG lasers on the SBS of ceramic brackets cemented to composite blocks. | 36 composite blocks were made and ceramic brackets were bonded to them, which were then irradiated with an Er:YAG or Er,Cr:YSGG laser. SBS, ARI and surface damage were assessed. | No significant differences in surface damage, ARI, or SBS were found between the irradiated groups and the control group. | The study did not demonstrate any reduction in the SBS of ceramic brackets with composite blocks following Er:YAG and Er,Cr:YSGG laser irradiation. |
| Mundethu [61] | Evaluation of the suitability of the Er:YAG laser for debonding ceramic brackets with a single pulse. | Debonding was performed on human teeth, then ARI and SEM images were evaluated. | 19 of 20 brackets were successfully debonded with one pulse. ARI was 3. SEM images showed no laser-related morphological changes. | The debonding process was due to thermomechanical ablation in the superficial part of the adhesive layer. |
| Tozlu [62] | Assessment of the time lag elapsed between lasing and shearing on debonding of ceramic brackets. | 100 ceramic brackets were detached from human teeth for various lengths of time after Er:YAG laser irradiation. SBS and ARI were assessed. | After laser irradiation, the SBS value was lower than in the control group. No significant difference was shown for ARI. | Debonding of ceramic brackets after 18 s using a 6 s Er-YAG laser scanning method is safe and suitable for clinical use. |
| Hoteit [63] | The objective was to assess the variations in enamel topography caused by various erbium laser settings applied during the debonding process. | Using scanning techniques, 180 bovine incisor teeth were randomly assigned to 15 experimental groups based on various Er:YAG settings. To determine the frequency of enamel microcrack formation and enamel loss, SBS testing was carried out following debonding, and stereomicroscopic and SEM investigations were carried out following the cleaning of the leftover adhesive. | Comparing the proportions of teeth with normal enamel topography in the control group to any of the Er:YAG groups revealed no statistically significant changes. | The enamel surface may change during ceramic bracket debonding if laser settings are not properly adjusted, according to the constraints of our investigation. |

| | | | | |
|---|---|---|---|---|
| Hamadah [64] | In order to develop safe and efficient procedures for debonding ceramic brackets, the research aims to assess the thermal impact of various Er:YAG laser pulse durations. | For orthodontic procedures, 45 premolars were removed and included in the sample. Each tooth was bonded to a ceramic bracket. 50 teeth for a 50 μs pulse, 50 teeth for a 100 μs pulse, and 15 teeth for a 300 μs pulse were included in the sample. Under the same air and water conditions, with the same pulse energy and repetition rate, all of the ceramic brackets were exposed to the Er:YAG laser for 6 seconds using the laser scanning method. | The findings demonstrated that there was no statistically significant difference in the tooth's temperature rise between pulse durations of 50, 100, and 300 μs. There was no statistically significant difference between 100 and 300 μs, although there was a statistically significant difference between 50 μs and both 100 and 300 μs in terms of the presence of adhesive materials. | When ceramic brackets are debonded using the laser scanning approach, Er: YAG pulse lengths of 100 and 300 μs are both preferable within the parameters of this investigation. |
| Nalbantgil [65] | In order to determine the most appropriate energy level for clinical application, this study compared the intrapulpal temperature change caused by various Er:YAG laser energy levels utilised during the debonding of ceramic brackets. | Bovine incisor teeth had 80 polycrystalline alumina brackets applied to them; these teeth were randomly assigned to four groups of 20. A control group was designated. An Instron Universal Testing equipment was used to debond the brackets in the research groups following laser exposure at energy levels of 2, 4, or 6 watts. To assess the debonding site, adhesive remnant index (ARI) scores were noted. A thermocouple was used to prepare 60 human premolar teeth at the same energy levels and in the same manner in order to measure intrapulpal thermal increase. | Examining the groups' debonding forces, intrapulpal temperature rises, and ARI revealed statistically significant differences between them. The average temperature for the 2 Watt, 4 Watt, and 6 Watt laser groups was noted. The control group's mean shear bond strength was between 21.35 and 3.43 megapascals (MPa), while the 2, 4, and 6 Watt laser groups' respective mean shear bond strengths were 8.79 and 2.47, 3.28 and 0.73, and 2.46 and 0.54 MPa. | The most effective and secure energy level to utilise is four watts, which uses an Er:YAG laser with a water cooling spray for six seconds using the scanning method to debond polycrystalline alumina brackets without causing any harmful temperature fluctuations at the debond sites or carbonisation effects. |
| Didem Nalbantgil [66] | Determine the duration of ER:YAG laser application to debond brackets without damaging the pulp | Teeth with ceramic brackets were divided into groups and exposed to laser radiation for different lengths of time( 3,6,9 sec.) | Laser irradiation of teeth caused an increase in pulp temperature(each group's temperature rise was below the 5.5°C benchmark). The temperature increased in proportion to the irradiation time. | Laser exposure times for bracket debonding were safe for the pulp in all study groups. |
| Oztoprak M. O. [67] | To examine a new technique for removing ceramic brackets using an | 60 bovine mandibular incisors were split into 2 groups. The Er:YAG laser (4.2W) applied in a scanning motion for 9 seconds was used in the test | Scanning method reduced the force needed to remove brackets. The laser-treated teeth retained | The Er:YAG laser scanning method proved to be an effective method for |

| | | | | |
|---|---|---|---|---|
| | Er:YAG laser with scanning motion. | group, while the control group received none. | more adhesive which indicates a safer debonding with lower risk of enamel damage. | removing ceramic brackets. |
| Alakuş-Sabuncuoğlu F. [68] | To examine the effectiveness of the Er:YAG laser at removing ceramic brackets and investigate its impact on the bonding force and remaining adhesive patterns. | Human's 20 mandibular incisors with attached polycrystalline brackets were splitted into 2 groups. The laser groups received Er:YAG (3W for 6sec) using a scanning method. Measurements of SBS and ARI were made. | The bonding strength was significantly lower in the laser group. Laser-treated teeth had over 50% of the adhesive residue on the tooth surface. No tooth damage was observed in either group. | The laser technique successfully weakened the bracket's bonding but remains more residues. |
| Rao [69] | Observation and analysis of changes on the enamel surface occurring as a result of laser debonding of metal and ceramic brackets using an Er, Cr:YSGG laser. | After debonding orthodontic brackets from human teeth using an Er,Cr:YSGG laser with different parameters, ARI was assessed and SEM images were taken. | After laser irradiation, ARI and enamel microdamage were smaller than in the control group. | The use of Er, Cr:YSGG laser for debonding orthodontic brackets provides patients with high-quality care with minimal damage after treatment. |
| Abdulaziz [70] | Compare the effects of a traditional approach and two distinct laser modes (circular and scanning) on the surface of the enamel following the debonding of orthodontic brackets. | Three groups were formed from the 66 removed premolars. Attaching the ceramic brackets to the teeth was done using light-cure composite resin. Specimens in Group I underwent conventional debonding with pliers, while specimens in Group 2 underwent circular motion debonding using Er, Cr: YSGG laser applications, and specimens in Group 3 underwent scanning motion debonding using Er, Cr: YSGG laser applications. Scanning electron microscopy was used for the evaluation of the enamel's microstructure, surface roughness following polishing, intra-pulpal temperature increase, and Adhesive Remnant Index (ARI). | Compared to the circular and scanning laser groups, the conventional debonding approach had a substantially greater proportion of adhesive residual index (ARI) ratings of 2 and 3. The ARI ratings of the circular and scanning laser groups did not differ significantly. Furthermore, in comparison to the group using the conventional procedure, the circular and scanning laser debonding methods produced a considerably larger proportion of enamel surface roughness (ESR) ratings of 0 and a lower proportion of ESR scores of 3. The circular and scanning laser approaches did not, however, significantly | Er, Cr: One method that shows promise for debonding ceramic brackets with little damage to the enamel surface is YSGG laser irradiation. The decreased intra-pulpal temperature increase makes the scanning laser approach more appealing. |

| | | | | |
|---|---|---|---|---|
| | | | differ in their ESR values. Finally, compared to the scanning laser group, the circular laser group's average intra-pulpal temperature was noticeably higher. | |
| Stein [71] | To investigate the temperature increase of the tooth pulp during the debonding of ceramic brackets using a diode laser with a wavelength of 445 nm. | Pulp fluid circulation was simulated in 18 human teeth with ceramic brackets. They were irradiated with a 445 nm diode laser. The temperatures in the pulp center and in the hard tissue of the tooth were measured at the beginning and end of the procedure. | The maximum temperature increase in the pulp was 2.23 degrees. | Removing ceramic brackets with a 445nm diode laser does not pose a threat to the vitality of the pulp. |
| Stein [72] | To investigate whether the bracket failure mode is changed by using diode laser 445nm prior to debonding. | Ceramic brackets bonded to 15 human teeth were irradiated with a 445nm diode laser, then the ARI was assessed and the enamel surface was examined under magnification and by SEM. | ARI was lower after laser irradiation than in the control group. Enamel damage was only visible on SEM in the control group. | Irradiation with a 445 nm diode laser before removing ceramic brackets significantly reduces the amount of remaining adhesive material, which helps reduce the risk of enamel damage. |
| Stein [73] | Evaluation of the effect of exposing ceramic brackets to a 445nm diode laser on SBS. | 30 ceramic brackets were bonded to human teeth, laser irradiation was applied, and SBS, ARI, and surface damage were assessed. | Both SBS and ARI were lower after laser irradiation than in the control group. No enamel damage was observed. | The use of a laser lowers the SBS value, reduces the risk of enamel damage and shortens the working time. |
| Yassaei [74] | This study aims to assess the pulpal temperature changes and enamel surface features of teeth following ceramic bracket debonding, either with or without laser light. | Using a diode laser (2.5 W, 980 nm) or conventional means, thirty polycrystalline brackets were glued to thirty undamaged removed premolars and subsequently debonded. Ten seconds of sweeping movement were used to apply the laser. The adhesive remnant index (ARI), lengths, and frequency of enamel cracks were examined between the groups following debonding. It was also measured how much the intrapulpal temperature increased. | None of the groups experienced an enamel fracture. When compared to traditional debonding, laser debonding significantly reduced the frequency and widths of enamel fissures. The intrapulpal temperature increase in the laser debonding group (1.46ºC) was much lower than the standard of 5.5ºC for every specimen. There was no discernible | Without heating the pulp, laser-assisted debonding of ceramic brackets may lower the chance of enamel deterioration. All debonding techniques, however, should be anticipated to result in some increases in the length and frequency of enamel fissures. |

| | | | | |
|---|---|---|---|---|
| | | | variation in the groups' ARI ratings. | |
| Feldon [75] | The efficiency of using a diode laser to debond ceramic brackets. | Both monocrystalline and polycrystalline ceramic brackets were attached to the maxillary central incisors of cows. For three seconds, brackets in the experimental groups were exposed to the diode laser.<br>At two laser energy levels—2 and 5 W per square centimeter—the shear bond strength and heat effects on the pulp chamber were evaluated. Significant variations in shear bond strength values were identified using analysis of variance (ANOVA). | The diode laser considerably ($P < 0.05$) reduced the shear bond strength with monocrystalline brackets but was ineffective with polycrystalline brackets. No groups evaluated showed appreciable variations in adhesive remnant index scores. | The debonding force needed for monocrystalline brackets was greatly reduced by diode laser use without appreciably raising the pulp chamber temperature. The amount of debonding force needed for polycrystalline brackets was not considerably reduced by diode lasers. |
| Ayşe Sena Kabaş Sarp [76] | The search for the optimal laser-based method for the removal of ceramic brackets without damage to the tooth. | Bovine teeth with ceramic brackets were irradiated with ytterbium fibre laser in continuous and modulated mode. The following parameters were evaluated during the study: debonding force, debonding time, pulp temperature increase. | In the experimental groups, the force and time required to remove the bracket was less than in the control group. In most of the experimental groups, the temperature increase was below the threshold (5.5°C). | The use of ytterbium fibre lasers for debonding orthodontic ceramic brackets makes this procedure faster and safer for the tooth. |
